# Human Biomonitoring of PCDDs, PCDFs, and PCBs in Women Living in a Southern Italy Hotspot Area

**DOI:** 10.3390/toxics13090730

**Published:** 2025-08-29

**Authors:** Roberto Miniero, Vittorio Abate, Annalisa Abballe, Tatiana Battista, Michele Conversano, Elena De Felip, Silvia De Luca, Anna Rita Fulgenzi, Nicola Iacovella, Anna Laura Iamiceli, Simona Insogna, Valentina Marra, Carmela Russo, Anna Maria Ingelido

**Affiliations:** 1 Unit of Human Exposure to Environmental Contaminants, Department of Environment and Health, Istituto Superiore di Sanità, 00161 Rome, Italy; vittorio.abate@iss.it (V.A.); annalisa.abballe@iss.it (A.A.); elena.defelip@iss.it (E.D.F.); silvia.deluca@iss.it (S.D.L.); annarita.fulgenzi@iss.it (A.R.F.); nicola.iacovella@iss.it (N.I.); annalaura.iamiceli@iss.it (A.L.I.); simona.insogna@asl.taranto.it (S.I.); valentina.marra@iss.it (V.M.); annamaria.ingelido@iss.it (A.M.I.); 2 Department of Prevention, Public health and Hygiene, Azienda Sanitaria Locale, 74100 Taranto, Italy; tatiana.battista@asl.taranto.it (T.B.); michele.conversano@asl.taranto.it (M.C.); carmela.russo@asl.taranto.it (C.R.)

**Keywords:** exposed/not exposed study, hotspot, breast milk, PCDDs, PCDFs, PCBs, Italy

## Abstract

Taranto is the main harbor in Southern Italy and one of the most industrialized cities in the country, largely due to the presence of a large industrial area that includes a major oil refinery, a cement plant, and the former ILVA steel factory, which is one of the largest steel plants in Europe. A human biomonitoring study was conducted on breast milk from two groups of women residing in areas with different levels of exposure to polychlorodibenzo-*p*-dioxins (PCDDs), polychlorodibenzofurans (PCDFs), and polychlorobiphenyls (PCBs). The study aimed to assess the differences in exposure between the two groups of general people, with one group classified as “exposed” and the other as “non-exposed”. Between 2015 and 2018, 150 breast milk samples were collected: 76 from the exposed group and 74 from the non-exposed group. A specific questionnaire was also administered to the donors. The data were analyzed using a robust regression approach. The results showed significant differences in the concentrations of all analyte classes between the two groups. The difference in concentration from the non-exposed to the exposed group was highly significant (TOT_TE_, 5.70 vs. 7.35 pgWHO-TE/g, PCDD + PCDF 3.34 vs. 4.53 pgWHO-TE/g, DL-PCB 2.35 vs. 2.80 pgWHO-TE/g; *p* << 0.05), with the most notable difference observed for the Σ_10_ (PCDFs) family (~37%). Additionally, two distinct theoretical exposure profiles were identified: one for women residing in urban peripheries and another for those living in city/town centers. Women in the peripheries were characterized by a profile of four to six chlorinated dioxin/furan congeners plus two PCB congeners, while women in the city centers exhibited a profile of six to eight chlorinated PCDD and PCDF congeners plus five to six chlorinated PCBs. Among women residing in urban peripheries, those living in the peripheries of Statte and Taranto showed the highest exposure levels. All the results appear to witness the highest exposure of the exposed women deriving from the steel plant of concern. In addition, the highest exposure levels for the analytical sum of Σ_6_ (NDL-PCBs) were found in women from a municipality classified as non-exposed: Ginosa (periphery).

## 1. Introduction

The town of Taranto, located in the Apulia region of southern Italy, serves as the primary harbor in Southern Italy and is one of the most industrialized cities in the country. This is largely due to the presence of a significant industrial area that includes a large refinery, a cement plant, and the ILVA steel plant, which is the largest steel production facility in Europe. The activities of the ILVA plant, which began in the early 1900s, have been identified as the primary source of extensive and long-lasting pollution in the Taranto area. This pollution is characterized by the release of heavy metals, polycyclic aromatic hydrocarbons, and dioxins into the environment.

Due to the presence of the former ILVA plant, as well as other emission sources—including both regulated and illegal waste disposal sites—Taranto has been classified as a high environmental risk area in Italy. Along with the neighboring municipality of Statte, it has been included in the list of Italian Polluted Sites (IPS) of national interest. Epidemiological surveillance in this IPS has been periodically conducted under the SENTIERI Project [1,2,3,4], which has documented numerous critical issues in the health profile of the local population.

The most recent update from the SENTIERI Project [4] confirms elevated risks of mortality and morbidity (hospitalization) in both genders for all causes, all cancers, and diseases of the cardiovascular, respiratory, and digestive systems. Excess risks were also observed for mortality and hospitalization related to conditions with sufficient or limited evidence of association with the emission sources in the area, such as lung cancer, pleural mesothelioma, and acute and chronic respiratory diseases. In addition to lung cancer and mesothelioma, increased cancer incidence was detected in several other sites of potential interest, including the liver, pancreas, melanoma, breast, kidney, bladder, and thyroid in males, and the stomach, liver, melanoma, uterus, breast, thyroid, and leukemia in females. Excess incidence risks were also observed in children (0–14 years) for non-Hodgkin lymphomas and soft tissue sarcomas, as well as in young adults (20–29 years) for thyroid cancer and germ cell tumors. Furthermore, an elevated risk of congenital anomalies was identified among live births. The documented health impairments in the IPS have prompted numerous public health initiatives aimed at assessing population exposure to pollutants of major concern and evaluating trends in exposure over time.

Dioxins, which include polychlorinated dibenzodioxins (PCDDs) and polychlorinated dibenzofurans (PCDFs), are organic pollutants of significant toxicological concern, primarily associated with industrial and metallurgical activities. The most important effects determined on human beings include skin problems like chloracne, liver damage, and potential impacts on the immune, reproductive, and endocrine systems [5]. Recently, a statistically significant positive association between a cumulative exposure to airborne dioxin exposure and chronic lymphocytic leukemia (CLL) and non-Hodgkin’s lymphoma (NHL) was found in a case–control study carried out in France [6]. The release of these substances into the environment has been significantly reduced in recent decades as a result of international regulatory measures. However, given the extremely persistent nature of dioxins and PCBs, infant exposure can occur even long after these substances have been released into the environment, especially in hot spots. For this reason, it is very important to continue monitoring human exposure, especially of sensitive groups, such as children and infants. Biomonitoring studies are widely regarded as the most reliable method for assessing human exposure to these contaminants as part of a comprehensive risk assessment strategy. The most widely used matrix for human biomonitoring of dioxins and PCBs is serum, but sampling is highly invasive. Breast milk sampling, on the other hand, is less invasive and offers the distinct advantage of being able to estimate infant exposure without subjecting them to direct sampling. Human biomonitoring data from women of reproductive age provide critical insights into potential impacts on female reproductive health because some reports suggest that exposure to low levels of dioxins in the environment may cause subtle delays in development and alterations in thyroid function to the children of mothers exposed during pregnancy [7]. Additionally, such data allow for the estimation of a child’s body burden of these contaminants during the early years of life, resulting from both in utero exposure and breastfeeding.

However, similar to trends observed in other countries, exposure to PCDDs, PCDFs, and polychlorinated biphenyls (PCBs) in Italy has been declining, with chemical-specific rates of decrease [8,9]. Considering this, in 2011, the Italian Ministry of the Environment incorporated a human biomonitoring study on maternal milk into the activities required of ILVA as part of the revision of the Integrated Pollution Prevention and Control (IPPC) permit, known in Italy as Autorizzazione Integrata Ambientale (AIA). ILVA commissioned the Istituto Superiore di Sanità (ISS) to conduct this study, which was carried out between 2015 and 2018 in collaboration with the Department of Prevention of the Local Health Authority (ASL) of Taranto.

## 2. Materials and Methods

### 2.1. Donor Selection and Recruitment

The Department of Prevention of the ASL of Taranto was responsible for donor recruitment and sample collection. Participants were selected based on their residential area: women residing in Taranto and Statte (referred to as the “exposed” group) and women residing in municipalities located more than 30 km from Taranto, considered to have background exposure levels (referred to as the “non-exposed” group). Inclusion criteria for donating mothers, all from the general population, were as follows: age between 20 and 40 years, local residency for at least 10 years, physiological childbirth, exclusive breastfeeding of one child, and primiparity. Between March 2017 and March 2018, 150 breast milk samples were collected, comprising 76 from the exposed group and 74 from the non-exposed group. During recruitment, particular attention was given to balancing the donors by age group, a critical parameter given the known influence of age on the body burden of persistent organic pollutants (POPs), such as those analyzed in this study. Each participant, assisted by an ASL operator, completed a questionnaire regarding lifestyle and dietary habits to support the interpretation of analytical data. Personal data were collected and stored in compliance with privacy protection legislation. The study was approved by the Local Ethics Committee of Brindisi (ASL BR (Independent Committee of Medical Ethics of Brindisi)).

### 2.2. Sample Collection

All participants received detailed instructions on the proper collection and storage of breast milk samples, which were to be collected between the fourth and eighth week postpartum, according to standardized milk sampling and analytical protocols provided by the WHO in collaboration with the UNEP [10]. Each donor was provided with a 100 mL contamination-free glass bottle and a manual breast pump (Artsana S.p.A., Como, Italy). The milk samples were stored at −20 °C until analysis.

### 2.3. Participating Institutions

The study was conducted by the Italian National Institute of Health (ISS), specifically the “Human Exposure to Environmental Contaminants” unit, in collaboration with the Department of Prevention of the ASL of Taranto. The ISS designed the study, developed written protocols for local medical professionals (including doctors, pediatricians, gynecologists, and midwives), prepared donor materials, performed contaminant analysis, and conducted exposure assessments. The Department of Prevention of the ASL of Taranto was responsible for donor recruitment, sample collection, and the selection of local healthcare facilities involved in the study. They also distributed informational materials, administered questionnaires and informed consent forms, collected and stored samples until shipment to the ISS, and collaborated with the ISS in communicating the results.

### 2.4. Chemical Analysis

Milk samples were stored under refrigeration until analysis. After thawing and homogenization, each sample (20 g) was spiked with internal standards containing PCDD, PCDF, and PCB congeners to be quantified, (obtained from Cambridge Isotope Laboratories, USA, and from Wellington Laboratories, Guelph, Canada). After, milk was subjected to liquid–liquid extraction using a mixture of ethyl ether, n-hexane, and methanol (20 mL each). The organic extract was purified by elution through a sodium sulfate column. The eluate was further treated and purified using the multicolumn DEXTech system (LCTech, DE). The fat content was determined gravimetrically from a fraction of the eluate. Quantification of persistent organic pollutants (POPs) was performed in accordance with the Environmental Protection Agency (EPA) methods 1613b [11] and 1668c [12]. High-resolution gas chromatography coupled with high-resolution mass spectrometry (HRGC-HRMS) was used for the analysis of PCDDs, PCDFs, and DL-PCBs, while low-resolution mass spectrometry (LRMS) was employed for NDL-PCBs.

### 2.5. Quality Assurance (QA)/Quality Control (QC)

Analytical reliability was ensured through the analysis of procedural blank samples and blind replicates. The acceptability limits for the blanks were adapted from Section 9.5.2 of EPA 1668C [12] and Section 6.5 of EU Regulation 644/17 [13]. Accuracy was evaluated using in-house reference matrices fortified with 13C-labeled internal standards at concentrations close to background levels. All results were within the set 20% variation limit. For the POPs analyzed, ^13^C-labeled IS recovery rates ranged from 60 to 120%. LOQ values ranged from 0.007 to 0.01 pg/g fw (fresh weight) for PCDDs and PCDFs, from 0.01 to 0.08 pg/g fw for non-ortho PCBs, and from 0.001 to 0.005 ng/g fw for mono-ortho and NDL-PCBs.

The analytical method was validated through participation in various national and international quality control studies and proficiency tests. The laboratory is accredited according to ISO/IEC 17025 standards [14] and regularly participates in international interlaboratory comparison exercises. 

### 2.6. Statistical Analysis

A targeted questionnaire was administered to participants to identify potential factors influencing the body burden of pollutants. The questionnaire collected data on personal, environmental, and dietary variables, which were initially checked for missing values, and their relative frequencies were estimated. Given the large number of variables, a statistical screening approach was employed to identify variables showing a significant difference (*p* ≤ 0.05) between the two groups using the Mann–Whitney U test. Subsequently, the selected variables were analyzed using a multiple linear robust regression approach (Stata 16, Stata Corp., College Station, TX, USA). The Variance Inflation Factor (VIF) was used to assess collinearity among independent variables. The final models were developed through an iterative process: starting with a regression model including all significant variables; variables were progressively eliminated based on their *p*-values until the model achieved maximum statistical reliability. Additionally, a *p*-value threshold of *p* ≥ 0.2 was applied to include non-significant variables in the final models, following the approach of Maldonado and Greenland [15]. The distribution of single congener data was analyzed only when the substance was quantified in at least 60% of the samples [16]. Further statistical analyses, including marginal means, marginal predicted choice probabilities, and adjusted predictions based on the previously fitted models, were conducted. These calculations were performed at fixed values of the covariates and averaged over the covariates of interest. This post-estimation strategy provides estimates that facilitate the interpretation of the results from a choice model (Stata 16).

## 3. Results

### 3.1. Participant Characteristics

The inclusion criteria for participants were met in both groups. The characteristics of the donors are summarized in Table 1. The most critical variables potentially influencing body burden were highly comparable between the two donor groups. In particular, the age distribution across different age classes, as well as between the donor groups, was well balanced. Given that age is a critical parameter influencing the body burden of persistent organic pollutants (POPs), this balance ensures strong comparability between the two groups. Among all variables related to food consumption, only the consumption of pork and dried fruits showed significant differences between the two groups (Table S1).

### 3.2. Levels of Selected Contaminants in Human Milk

Milk concentrations of PCDDs, PCDFs, and PCBs detected in the exposed and not exposed group were comparable to data from studies conducted in Italy in the same period on milk samples from groups of donors residing in industrial areas and in areas at background exposure, respectively [17,18]. The results of multiple robust regression analyses are presented in Table 2, Table 3 and Table 4.

These tables display the adjusted means and 95% confidence intervals for the categories of predictors of interest, calculated from the predictions of the relevant models at fixed values of the covariates. The corresponding regression models are detailed in Table S2, with highly significant associated-probabilities (*p* < 0.001). Table 2 highlights the differences between the exposed and non-exposed populations including the chemical groups as well as the pertinent congeners. Table 3 compares the differences across various areas of the cities/towns (rural areas, urban peripheries, and city/town centers) for the chemical groups and the congeners as above. Table 4 examines the differences across participant age classes, while Table 5 describes the mean and median concentrations of PCDD + PCDF (pg/g lb), TOT-TEQ (pg/g lb), DL-PCB (pg/g lb), PCDD (pg/g lb), PCDF (pg/g lb), and NDL-PCB (ng/g lb) of the cities/town involved in the research.

## 4. Discussion

In Table 2, the differences between the non-exposed and exposed groups for all chemical families (*p* < 0.001) show a general increase, with variability depending on the chemical family. Specifically, the increases range from 14.7% to 33.6% for the chemical families of Σ_7_ (PCDDs) and Σ_10_ (PCDFs), respectively. Among all chemical families in Table 2, the highest increase was observed for the Σ_10_ (PCDFs) group, which is a typical indicator of exposure to industrial sources [19]. Table 3 evaluates the same chemical families but categorizes them by “zone”: rural areas, urban peripheries, and city/town centers. The highest increases in WHO-TE values were detected in women from urban peripheries, while one of the four chemical families showed the highest increase in women from city/town centers. This apparent discrepancy suggests that the slight increase in values in urban peripheries may be due to the presence of congener (s) with high toxicological potential. Table 4 reports exposure levels according to the age of the participants. The highest increases were observed for Σ_6_NDL-PCBs (~40%) and DL-PCBs (~36%), while no significant increase was observed for the Σ_7_ (PCDDs) and Σ_10_ (PCDFs) families. Among the WHO-TE values, the highest increase was observed for TOT_TEQ_ (~21%). This confirms that in primiparous pregnant women, as the age increases, so does the body’s dioxin body burden. In turn, this would imply that a higher body burden could be transferred to offspring during lactation [20].

The congeners that best characterize the exposed group, based on those showing the highest differences (6th column of Table 5), are 2,3,7,8-T_4_CDD; 1,2,3,4,7,8-H_6_CDF; 1,2,3,6,7,8-H_6_CDF; 2,3,4,6,7,8-H_6_CDF; 1,2,3,4,6,7,8-H_7_CDF/g; and P_5_CB99. All these congeners show an increase of ≥34.4% compared with the non-exposed group. These findings suggest the presence of at least two distinct exposure profiles within the study population: one characterizing the exposed individuals and another characterizing the non-exposed individuals. Further, the above-mentioned four furan congeners were detected in air samples collected above a large iron and steel plant located in Anshan city, northeast China. The plant is one of the largest iron and steel plants in China, with a long production history of over 50 years, and has an annual iron and steel production capacity of 32 million tons [21]. Further details are provided in Table 3, which highlights the congeners that characterize women residing in three different areas: rural, peripheral, and city centers. Women living in rural areas consistently exhibit the lowest levels of exposure, while those in peripheral areas and city centers show distinct exposure patterns. The chemicals that specifically mark women living in urban peripheries (i.e., those showing a significant concentration increase, *p* ≤ 0.05) are 2,3,7,8-T_4_CDD; 1,2,3,7,8-P_5_CDD; 1,2,3,6,7,8-H_6_CDD; 1,2,3,7,8,9-H_6_CDD; 2,3,4,7,8-P_5_CDF; 2,3,4,6,7,8-H_6_CDF; PCB169; and H_7_CB189. In contrast, the congeners characterizing women living in city centers are 1,2,3,4,7,8-H_6_CDD; O_8_CDD; 1,2,3,4,7,8-H_6_CDF; 1,2,3,4,6,7,8-H_7_CDF; PCB105; PCB114; PCB118; PCB123; HCB157; HCB167; PCB99; HCB138; and HCB153. These results indicate that different exposure sources characterize urban peripheries and city centers. Specifically, it is possible to delineate two distinct exposure patterns: one primarily consisting of four to six chlorinated dioxin/furan congeners plus two PCBs for women living in peripheries, and another consisting of six to eight chlorinated PCDD and PCDF congeners plus five to six chlorinated PCBs for women living in city centers. However, considering the chemical groups listed in Table 2 and the municipalities involved (Table 5), the exposed group resides in the peripheries of Taranto and Statte, as well as in the center of Taranto. Women in these areas/municipalities exhibit the highest exposure levels to PCDD + PCDF, TOT_TEQ_, DL-PCB, Σ_7_ (PCDDs), and Σ_10_ (PCDFs) among the sampled peripheries and city centers (Table 5).

These findings align with the results presented in Table 2, where the exposed women show an increase in the same chemical groups ranging from 16.1% to 33.6%. This suggests that, among the possible exposure profiles, the maternal milk of women living in these areas displays specific characteristics: (i) The highest levels of toxicological potential values are likely due to the elevated levels of 2,3,7,8-T_4_CDD detected in the exposed group (34.4%, Table 2); (ii) two theoretically distinct exposure profiles differentiate women from urban peripheries and city centers (Table 3). The zones of Statte and Taranto, which are the most polluted among those listed in Table 5, appear to be the best characterized by these profiles. Additionally, among the municipalities not included in the exposed group, women living in the periphery of Ginosa exhibit the highest exposure to ∑_6_NDL-PCBs. However, the sample size for each municipality is small, so these findings require further confirmation. The women living in the exposed area are impacted by the dumping zone of the steel plant ILVA, which is currently under controlled administration but was under the ownership of ArcelorMittal at the time of sampling. It is well-documented that thermal metallurgical processes generate significant volumes of off-gases. Chlorinated and volatile organic compounds are present in the feedstock, and as a result, the off-gases from such processes typically contain dioxins [22,23]. For instance, lower chlorinated PCDF congeners such as 2,3,7,8-T_4_CDF (2,3,7,8-tetrachlorodibenzofuran); 1,2,3,7,8-P_5_CDF (1,2,3,7,8-Pentachlorodibenzofuran); and 2,3,4,7,8-P_5_CDF (2,3,4,7,8-pentachlorodibenzofuran) were detected in Portuguese siderurgical units [24]. However, the congener content of off-gases appears to vary. In the atmosphere surrounding a steel plant in Northeast China, only 1,2,3,4,6,7,8-H_7_CDD (1,2,3,4,6,7,8-heptachlorodibenzo-p-dioxin) and OCDD (octachlorodibenzo-*p*-dioxin) were detected for dioxins, while most PCDF congeners were identified, except for 1,2,3,7,8,9-H_6_CDF (1,2,3,7,8,9-hexachlorodibenzofuran) and 1,2,3,4,7,8,9-H_7_CDF (1,2,3,4,7,8,9-heptachlorodibenzofuran) [21].

## 5. Conclusions

Contaminant concentrations in the exposed and not exposed group from this study were almost comparable with data from studies on human milk conducted in Italy in the same period on population groups residing in industrial areas and in areas at background exposure [17,18]. The difference in contaminant concentration between the two groups of donors, classified as exposed and non-exposed, was confirmed. Milk concentrations of all the considered compound families were significantly higher in the exposed women. The differences between the adjusted mean values in the two groups ranged from approximately 16% to 34%, depending on the chemical family. The largest difference was attributed to the Σ_10_ (PCDFs) family, indicating an industrial source of exposure. Indeed, the exposure profile of the exposed women was markedly different from that of the non-exposed group. The congeners that predominantly characterized the exposed women were 2,3,7,8-T_4_CDD; 1,2,3,4,7,8-H_6_CDF; 1,2,3,6,7,8-H_6_CDF; 2,3,4,6,7,8-H_6_CDF; 1,2,3,4,6,7,8-H_7_CDF/g; and PCB99. Additionally, distinct exposure profiles were identified for urban peripheries and city/town centers. Among the peripheries, Taranto and Statte, where the participating women were classified as exposed, exhibited the highest WHO-TE values in the sampling panorama. This was primarily due to a 34% increase in exposure to 2,3,7,8-T_4_CDD, the congener with the highest toxicological potential. This significant difference between the two groups is clearly attributable to the proximity of the exposed group to the industrial plant. Among the non-exposed towns included in this study, participants residing in Ginosa showed the highest levels of Σ_6_NDL-PCBs. Another noteworthy finding is that the transfer of contaminants to offspring through maternal milk increases with the age of the primiparous woman. This transfer also depends on the area of residence and on the chemicals. However, the chemicals showing the highest concentration are the Σ_6_NDL-PCBs (69.6 ng/g, Table S2). Overall, these conclusions should be interpreted in light of two key factors: (i) it has long been recognized that, despite chemical contamination [25], breast milk remains an essential food for newborns; and (ii) unpublished findings [26] suggest that exposure to the contaminants of interest in this area decreased by approximately 1.33 times over three consecutive studies conducted between 2011 and 2017. These data have been made available for the Italian Ministry of the Environment. Because of the results of the studies conducted to measure the levels of contaminants in the area impacted by the industry, Italian policy makers have stated a series of measures to contain emissions of the former ILVA plant.

## Figures and Tables

**Table 1:** Main parameters characterizing the two exposure groups.

Parameter	Exposed		Not Exposed	
N	76		74	
	Mean value	Range	Mean value	Range
Maternal age (years)	31.7	25–40	31.8	25–40
Body Mass Index (BMI)	25.1	18.3–34.3	24.5	19.1–38.3
Collection week	4.96	4.0–8.0	5.12	4.0–8.0
Smoking habits:				
bef. pregnancy (y/n)	34		24	
curr. smokers (y/n)	15%		5.4%	

**Table 2:** Adjusted means (pg or ng/g lb) (concentrations expressed on lipid base, lb) calculated from the predictions of the pertinent regression models (Table S2) for the categories of the variable “exposed/not exposed” at fixed values of the covariates. In the sixth column, there is the difference in percentage between the two adjusted means.

Chemical	Not Exposed		Exposed		Difference
	Mean	IC95	Mean	IC95	%
TOT_TE_, pgWHO-TE/g lb	5.70	5.23–6.15	7.35	6.86–7.83	22.4
PCDD + PCDF, pgWHO-TE/g lb	3.34	3.07–3.61	4.53	4.26–4.81	26.3
DL-PCB, pgWHO-TE/g lb	2.35	2.11–2.58	2.80	2.55–3.06	16.1
PCDD pg/g lb	24.4	21.1–27.5	28.6	25.8–31.2	14.7
PCDF pg/g lb	8.36	7.60–9.12	12.6	11.8–13.3	33.6
DL-PCB pg/g lb	8370	7663–9076	11,085	10,102–12,067	24.5
Σ_6_NDL-PCBs ng/g lb	48.6	43.9–53.1	58.7	53.3–63.9	17.2
TCDDpg/g lb	0.425	0.379–0.471	0.648	0.593–0.701	34.4
1,2,3,7,8-P_5_CDD pg/g lb	1.075	0.974–1.18	1.29	1.19–1.38	16.7
1,2,3,4,7,8-H_6_CDD pg/g lb	0.484	0.444–0.523	0.595	0.542–0.648	18.7
1,2,3,6,7,8-H_6_CDD pg/g lb	1.98	1.80–2.15	2.28	2.11–2.45	13.2
1,2,3,7,8,9-H_6_CDD pg/g lb	0.458	0.413–0.503	0.579	0.507–0.650	20.9
2,3,4,7,8-P_5_CDF pg/g lb	3.91	3.59–4.23	5.57	5.22–5.91	29.8
1,2,3,4,7,8-H_6_CDF pg/g lb	1.13	1.02–1.24	1.73	1.59–1.86	34.7
1,2,3,6,7,8-H_6_CDF pg/g lb	1.15	1.04–1.27	1.80	1.68–1.93	36.1
2,3,4,6,7,8-H_6_CDF pg/g lb	0.589	0.509–0.669	0.956	0.842–1.07	38.4
1,2,3,4,6,7,8-H_7_CDF pg/g lb	0.593	0.423–0.763	0.975	0.824–1.12	39.2
PCB126 pg/g lb	16.5	14.6–18.3	21.1	19.0–23.2	21.8
PCB105 pg/g lb	775	695–856	1042	932–1152	25.6
PCB114 pg/g lb	216	198–235	262	235–290	17.6
PCB118 pg/g lb	4317	3909–4726	5765	5207–6323	25.1
PCB123 pg/g lb	54.6	49.1–60.1	74.5	67.0–82.0	26.7
HCB156 pg/g lb	1951	1745–2158	2355	2076–2634	17.2
HCB157 pg/g lb	393	350–435	466	412–521	15.7
HCB167 pg/g lb	604	549–659	801	719–883	24.6
HCB189 pg/g lb	166	149–182	196	169–224	15.3
PCB99 ng/g lb	2.68	2.37–2.99	4.18	3.77–4.59	35.9
HCB138 ng/g lb	12.0	10.9–13.2	14.3	13.1–15.5	16.1
HCB153 ng/g lb	23.2	25.5–30.8	28.1	25.5–30.8	17.4

**Table 3:** In this table there are the adjusted means (pg or ng/g lb) (concentrations expressed on lipid base, lb) calculated from the predictions of the pertinent regression models (Tables S2 and S3) for the categories of the variable “zone” at fixed values of the covariates.

Chemical	Collection Areas		
	Rural Areas	Urban Peripheries	City/Town Centers
TOT_TE_, pgWHO-TE/g lb	5.65	6.82	6.68
PCDD + PCDF, pgWHO-TE/g lb	3.49	4.11	3.99
DL-PCB, pgWHO-TE/g lb	2.15	2.70	2.67
PCDD/g lb	22.2	26.3	29.3
PCDF/g lb	8.73	10.6	11.3
DL-PCB/g lb	8012	9999	10,572
∑6NDL-PCBsng/g lb	44.1	55.3	57.2
pgTCDD/g lb	0.486	0.575	0.517
1,2,3,7,8-P_5_CDD/g lb	1.07	1.23	1.18
1,2,3,4,7,8-H_6_CDD/g lb	0.457	0.560	0.563
1,2,3,6,7,8-H_6_CDD/g lb	1.87	2.23	2.15
1,2,3,7,8,9-H_6_CDD/g lb	0.426	0.556	0.524
O_8_CDD/g lb	16.2	19.1	22.1
2,3,4,7,8-P_5_CDF	4.17	4.90	4.89
1,2,3,4,7,8-H_6_CDF	1.24	1.45	1.51
2,3,4,6,7,8-H_6_CDF	0.609	0.820	0.814
1,2,3,4,6,7,8-H_7_CDF	0.567	0.661	1.10
PCB126/g lb	15.7	19.2	20.4
PCB169/g lb	11.3	14.0	13.6
PCB105/g lb	745	913	1008
PCB114/g lb	202	246	254
PCB118/g lb	4252	5172	5369
PCB123/g lb	54.1	66.1	69.2
HCB156/g lb	1754	2266	2267
HCB157/g lb	355	450	453
HCB167/g lb	580	730	744
HCB189/g lb	149	193	188
PCB99/g lb	2.72	3.34	4.11
HCB138/g lb	11.1	13.3	14.2
HCB153/g lb	21.0	26.5	27.4

**Table 4:** Adjusted means (concentrations expressed on lipid base, lb) calculated from the predictions of the pertinent regression models (Table S2) for the categories of the variable “age” at fixed values of the covariates.

Chemicals							
Age (y)	TOT_TE_, _pgWHO-TE/g lb_	PCDD + PCDF, _pgWHO-TE/g lb_	DL-PCB, _pgWHO-TE/g lb_	PCDDs, _pg/g lb_	PCDFs, _pg/g lb_	DL-PCBs, _pg/g lb_	Σ_6_NDL-PCBs, _ng/g lb_
25–28	5.72	3.56	2.15	26.1	9.62	7825	40.0
29–30	6.22	3.80	2.42	25.0	10.5	8880	51.0
31–32	6.43	3.81	2.61	29.2	9.98	9233	53.6
33–36	7.19	4.30	2.89	25.8	11.1	11,473	62.2
37–40	7.19	4.37	2.94	25.6	10.7	12,192	66.0

**Table 5:** Mean and median concentrations of PCDD + PCDF (pg/g lb), TOT-TEQ (pg/g lb), DLPCB (pg/g lb), PCDD (pg/g lb), PCDF (pg/g lb), and NDL-PCB (ng/g lb) (concentrations expressed on lipid base, lb) detected in the participants inform the different areas of the human settlements taken into consideration. In bold, there are the areas of residence of the exposed group.

	n	PCDD + PCDF		TOT_TEQ_		DL-PCB		Σ_7_(PCDDs)		Σ_10_(PCDFs)		Σ_6_(NDL-PCBs)	
		Mean	Median	Mean	Median	Mean	Median	Mean	Median	Mean	Median	Mean	Median
Avetrana center	4	3.82	4.26	6.60	6.50	2.78	2.25	56.2	49.3	12.2	11.9	39.8	51.1
Avetrana rural area	3	2.19	2.46	3.57	4.00	1.37	1.50	19.1	20.7	4.97	5.0	29.7	25.8
Castellaneta center	6	3.59	3.47	5.68	5.60	2.12	2.05	19.0	18.2	9.80	8.95	48.2	46.0
Ginosa center	5	2.71	2.67	5.38	5.50	2.66	2.80	26.3	18.5	7.00	6.9	45.7	48.1
Ginosa perip.	4	4.57	4.60	7.38	7.25	2.78	2.60	26.9	23.8	9.68	9.8	80.9	73.2
Laterza center	8	4.00	3.98	6.93	6.70	2.91	2.70	30.2	20.6	10.8	9.95	56.5	58.6
Laterza perip.	5	3.11	3.11	5.68	5.50	2.56	2.60	22.2	19.2	7.22	7.9	39.8	43.6
Manduria perip.	14	3.23	3.29	5.56	5.75	2.32	2.25	23.0	20.1	7.32	6.8	41.1	47.3
Manduria rural area	3	2.28	2.12	3.90	2.90	1.63	0.80	21.9	23.8	6.03	6.2	30.0	31.1
M. Franca center	10	3.15	3.18	5.43	5.45	2.28	2.20	18.9	16.2	8.58	7.9	53.9	55.9
M. Franca perip	7	4.25	3.89	7.11	7.00	2.89	2.80	26.6	26.5	10.9	9.3	62.7	63.8
Maruggio	3	2.49	2.34	4.13	3.70	1.60	1.50	22.5	24.1	7.40	7.8	19.8	22.1
Statte perip	5	4.70	4.31	7.84	7.70	3.16	3.40	28.4	22.7	13.1	11.2	54.7	61.1
Statte rural area	4	3.87	3.92	6.10	6.00	2.20	2.15	30.2	27.0	10.8	10.6	36.3	38.6
Taranto center	16	4.73	4.82	7.64	8.10	2.90	3.10	31.0	29.7	13.2	12.1	68.6	65.4
Taranto perip	34	4.75	4.51	7.70	7.45	2.96	3.00	29.0	28.3	13.0	12.5	59.5	59.7
Taranto rural area	17	4.02	3.75	6.36	5.80	2.34	2.20	22.2	22.4	10.8	10.6	46.2	48.6

## Data Availability

The Taranto Local Health Authority retains records of consent in both paper and electronic formats, in compliance with Articles 7 and 23 of Legislative Decree No. 196/03. Individual data cannot be shared openly to protect study participant privacy.

## Supplementary Materials

The following supporting information can be downloaded at: https://www.mdpi.com/article/10.3390/toxics13090730/s1, Table S1: Differences and missing rates of the questionnarie variables between the two exposure groups determined by the Mann-Whitney test; Table S2: Regression final models: main predictors for the dependent variables (congeners); Table S3: Regression final models: main predictors for the dependent variables (chemicals).
