# Supplementary material for: Human Biomonitoring of PCDDs, PCDFs, and PCBs in Women Living in a Southern Italy Hotspot Area
Source: Toxics. 2025 Aug 29;13(9):730. doi: 10.3390/toxics13090730 (PMC12473570; doi:10.3390/toxics13090730)
Supplement: Supplementary file 1 [file toxics-13-00730-s001.zip › toxics-3802983-supplementary.pdf]

Variable	P	n (missing)
Sampling Month	0.603	3
Zone	0.006	32
Past Smoker	0.274	92
Current smoker	1.000	87
Waste Treatment	0.000	76
Traffic	0.868	4
Industry	0.000	\
Milk consumption	0.110	20
Yoghurt consumption	0.549	69
Cereals consumption	0.877	\
Fresh cheese consumption	0.547	5
Seasoned cheese consumption	0.453	11
Eggs consumption	0.212	19
Beef consumption	0.790	40
Pig consumption	0.080	70
Chiken consumption	0.449	11
Cold cuts consumption	0.976	10
Fish consumption	0.351	16
Crustaceans consumption	0.132	121
Vegetable oil consumption	0.484	6
Animal fat consumption	0.160	94
Fruit consumption	0.372	4
Dry fruit consumption	0.056	77
Legumes consumption	0.129	23
Raw vegetables consumption	0.179	7
Cake, sweets consumption	0.256	8

Table S1. Differences and missing rates of the questionnarie variables between the two exposure groups determined by the Mann-Whitney test.

Table S2. Regression final models: main predictors for the dependent variables (congeners)

```
Linear regression                               Number of obs     =        145
                                                F(10, 134)        =       7.60
                                                Prob > F          =     0.0000
                                                R-squared         =     0.3097
                                                Root MSE          =     .21097

------------------------------------------------------------------------------------
                  |               Robust
       t4cdd2378 |      Coef.   Std. Err.      t    P>|t|     [95% Conf. Interval]
-----------------+------------------------------------------------------------------
            expo |   .2303579   .0386947     5.95   0.000     .1538266    .3068893
                 |
         TRAFFIC |
               2 |   .0420246   .0525819     0.80   0.426    -.0619732    .1460224
               3 |   .0481182   .0587293     0.82   0.414    -.0680381    .1642744
               4 |   -.032553   .0546596    -0.60   0.552    -.1406601    .0755541
                 |
```

Linear regression

Number of obs	=	148
F(7, 140)	=	10.01
Prob > F	=	0.0000
R-squared	=	0.2898
Root MSE	=	.20943

t4cdd2378	Coef.	Robust Std. Err.	t	P>|t|	[95% Conf.	Interval]
expo	.2222409	.0374569	5.93	0.000	.1481866	.2962951
age						
2	.053259	.0548866	0.97	0.334	-.0552547	.1617727
3	.001222	.057958	0.02	0.983	-.1133642	.1158081
4	.1091835	.046896	2.33	0.021	.0164676	.2018993
5	.0815258	.0707301	1.15	0.251	-.0583114	.221363

	Coef.	Std. Err.	t	P>|t|	[95% Conf.	Interval]
zone						
periphery	.0894393	.0470333	1.90	0.059	-.0035481	.1824267
center	.0313295	.0476966	0.66	0.512	-.0629692	.1256282
_cons	.3264603	.0477733	6.83	0.000	.2320099	.4209107

. margin age

Predictive margins Number of obs = 148
Model VCE : Robust

Expression : Linear prediction, predict()

	Margin	Delta-method Std. Err.	t	P>|t|	[95% Conf.	Interval]
age_new						
1	.491153	.0392867	12.50	0.000	.4134811	.5688249
2	.544412	.0380514	14.31	0.000	.4691824	.6196417
3	.492375	.0425753	11.56	0.000	.4082013	.5765486
4	.6003365	.0259129	23.17	0.000	.5491052	.6515677
5	.5726788	.0562804	10.18	0.000	.4614095	.6839481

. margin zone

Predictive margins Number of obs = 148
Model VCE : Robust

Expression : Linear prediction, predict()

	Margin	Delta-method Std. Err.	t	P>|t|	[95% Conf.	Interval]
zone						
rural	.486105	.0371208	13.10	0.000	.4127152	.5594948
periphery	.5755443	.028268	20.36	0.000	.519657	.6314317
center	.5174345	.0262979	19.68	0.000	.4654421	.5694269

Linear regression

Number of obs	=	145
F(10, 134)	=	4.29
Prob > F	=	0.0000
R-squared	=	0.1916
Root MSE	=	.41922

p5cdd12378	Coef.	Robust Std. Err.	t	P>|t|	[95% Conf.	Interval]

expo	.2455426	.0745203	3.29	0.001	.0981545	.3929308
TRAFFIC						
2	.0469768	.0948676	0.50	0.621	-.1406548	.2346085
3	.1706499	.168494	1.01	0.313	-.1626018	.5039016
4	-.126053	.0826035	-1.53	0.129	-.2894284	.0373224
age						
2	.0746781	.1113429	0.67	0.504	-.1455389	.294895
3	.1232874	.097018	1.27	0.206	-.0685972	.315172
4	.2762225	.0929399	2.97	0.004	.0924035	.4600416
5	.3835701	.1318071	2.91	0.004	.1228786	.6442616
zone						
periphery	.1356435	.0862661	1.57	0.118	-.0349757	.3062628
center	.1423208	.0927803	1.53	0.127	-.0411825	.325824
_cons	.7873406	.0986493	7.98	0.000	.5922294	.9824518

```
Linear regression                               Number of obs     =        148
                                                F(7, 140)         =       4.16
                                                Prob > F          =     0.0003
                                                R-squared         =     0.1593
                                                Root MSE          =     .41898
```

p5cdd12378	Coef.	Robust Std. Err.	t	P>|t|	[95% Conf.	Interval]
expo	.2154195	.0702873	3.06	0.003	.0764578	.3543812
age						
2	.0914715	.1099744	0.83	0.407	-.1259538	.3088969
3	.1530431	.1026641	1.49	0.138	-.0499294	.3560156
4	.2896353	.0929372	3.12	0.002	.1058935	.4733772
5	.3306251	.1216434	2.72	0.007	.0901297	.5711206
zone						
periphery	.1613574	.0862788	1.87	0.064	-.0092204	.3319352
center	.1087397	.0874193	1.24	0.216	-.0640929	.2815723
_cons	.8006694	.0979887	8.17	0.000	.6069405	.9943984

. margin age

Predictive margins   Number of obs = 148

Model VCE : Robust

Expression : Linear prediction, predict()

	Margin	Delta-method Std. Err.	t	P>|t|	[95% Conf.	Interval]
age						
1	1.021064	.067751	15.07	0.000	.8871165	1.155011
2	1.112535	.0860955	12.92	0.000	.9423199	1.282751
3	1.174107	.0791572	14.83	0.000	1.017609	1.330605
4	1.310699	.0672873	19.48	0.000	1.177669	1.44373
5	1.351689	.096255	14.04	0.000	1.161388	1.54199

. margin zone

Predictive margins Number of obs = 148

Model VCE : Robust

Expression : Linear prediction, predict()

	Margin	Delta-method Std. Err.	t	P>|t|	[95% Conf.	Interval]
zone						
rural	1.07323	.0651355	16.48	0.000	.9444541	1.202007
periphery	1.234588	.0551076	22.40	0.000	1.125637	1.343539
center	1.18197	.0579792	20.39	0.000	1.067342	1.296598

```
Linear regression                               Number of obs     =        145
                                                F(10, 134)        =       3.04
                                                Prob > F          =     0.0017
                                                R-squared         =     0.1494
                                                Root MSE          =     .20546
```

h6cdd123478	Coef.	Robust Std. Err.	t	P>|t|	[95% Conf.	Interval]
expo	.1217838	.0341276	3.57	0.000	.0542853	.1892822
TRAFFIC						
2	.0111318	.0509996	0.22	0.828	-.0897364	.1120001
3	.0819573	.0670155	1.22	0.223	-.0505877	.2145024
4	-.0014713	.0480415	-0.03	0.976	-.096489	.0935464
age						
2	.0761519	.0707525	1.08	0.284	-.0637843	.216088
3	.045449	.0403372	1.13	0.262	-.0343309	.1252289
4	.086867	.0380041	2.29	0.024	.0117014	.1620325
5	.1259081	.0689564	1.83	0.070	-.0104756	.2622919

zone						
periphery	.0894648	.0379762	2.36	0.020	.0143545	.1645752
center	.1066223	.0403137	2.64	0.009	.0268889	.1863557
_cons	.3300299	.0417375	7.91	0.000	.2474803	.4125794

Linear regression

Number of obs	=	148
F(7, 140)	=	4.00
Prob > F	=	0.0005
R-squared	=	0.1368
Root MSE	=	.2026

h6cdd123478	Coef.	Robust Std. Err.	t	P>|t|	[95% Conf.	Interval]
expo	.1113834	.0335561	3.32	0.001	.0450413	.1777256
age						
2	.0824386	.0652509	1.26	0.209	-.046566	.2114432
3	.0541003	.0418331	1.29	0.198	-.028606	.1368065
4	.0929117	.0389343	2.39	0.018	.0159364	.1698869
5	.1179613	.0645753	1.83	0.070	-.0097074	.2456301
zone						
periphery	.1025411	.0386892	2.65	0.009	.0260505	.1790317
center	.1056331	.039644	2.66	0.009	.0272548	.1840115
_cons	.3373224	.0383966	8.79	0.000	.2614102	.4132346

. margin age

Predictive margins — Number of obs = 148

Model VCE : Robust

Expression : Linear prediction, predict()

	Margin	Delta-method Std. Err.	t	P>|t|	[95% Conf.	Interval]
age						
1	.4765462	.0294995	16.15	0.000	.4182241	.5348682
2	.5589848	.0589903	9.48	0.000	.4423577	.6756118
3	.5306464	.0303509	17.48	0.000	.470641	.5906519
4	.5694578	.0257992	22.07	0.000	.5184513	.6204643
5	.5945075	.056121	10.59	0.000	.4835533	.7054617

. margin zone

Predictive margins   Number of obs = 148
Model VCE : Robust

Expression : Linear prediction, predict()

	Delta-method Std. Margin	Err.	t	P>|t|	[95% Conf.	Interval]
zone						
rural	.4577611	.0271767	16.84	0.000	.4040313	.5114909
periphery	.5603022	.0259366	21.60	0.000	.5090242	.6115802
center	.5633942	.0285937	19.70	0.000	.5068629	.6199256

Linear regression

Number of obs = 145
F(10, 134) = 4.36
Prob > F = 0.0000
R-squared = 0.2395
Root MSE = .75624

h6cdd123678	Coef.	Robust Std. Err.	t	P>|t|	[95% Conf.	Interval]
expo	.3592583	.136215	2.64	0.009	.0898487	.6286678
TRAFFIC						
2	-.0947983	.1668962	-0.57	0.571	-.4248899	.2352932
3	.1933749	.2423968	0.80	0.426	-.2860437	.6727935
4	-.1292566	.196006	-0.66	0.511	-.5169224	.2584093
age						
2	.2850717	.2167205	1.32	0.191	-.1435637	.7137071
3	.3459783	.1776694	1.95	0.054	-.0054209	.6973774
4	.7684208	.1813739	4.24	0.000	.4096947	1.127147
5	.9247842	.2690534	3.44	0.001	.3926435	1.456925
zone						
periphery	.3465629	.1467577	2.36	0.020	.0563018	.636824
center	.3274334	.1460064	2.24	0.027	.0386583	.6162085
_cons	1.272285	.1763595	7.21	0.000	.9234769	1.621094

Linear regression

Number of obs = 148
F(7, 140) = 5.82
Prob > F = 0.0000
R-squared = 0.2282
Root MSE = .74639

| | Robust

h6cdd123678	Coef.	Std. Err.	t	P>|t|	[95% Conf.	Interval]
expo	.3057105	.1222387	2.50	0.014	.0640381	.5473829
age						
2	.3182974	.2103488	1.51	0.132	-.0975735	.7341684
3	.393293	.1815896	2.17	0.032	.0342807	.7523053
4	.7776884	.1791266	4.34	0.000	.4235455	1.131831
5	.8924001	.2530633	3.53	0.001	.3920804	1.39272
zone						
periphery	.3552005	.14128	2.51	0.013	.0758824	.6345186
center	.2791326	.1409497	1.98	0.050	.0004676	.5577976
_cons	1.273057	.1724555	7.38	0.000	.9321035	1.614011

. margin age

Predictive margins Number of obs = 148
Model VCE : Robust

Expression : Linear prediction, predict()

	Margin	Delta-method Std. Err.	t	P>|t|	[95% Conf.	Interval]
age						
1	1.685994	.1435151	11.75	0.000	1.402257	1.969731
2	2.004291	.1549524	12.93	0.000	1.697942	2.310641
3	2.079287	.11289	18.42	0.000	1.856097	2.302477
4	2.463682	.1134399	21.72	0.000	2.239406	2.687959
5	2.578394	.1996502	12.91	0.000	2.183675	2.973113

. margin zone

Predictive margins Number of obs = 148
Model VCE : Robust

Expression : Linear prediction, predict()

	Margin	Delta-method Std. Err.	t	P>|t|	[95% Conf.	Interval]
zone						
rural	1.875768	.095961	19.55	0.000	1.686048	2.065488
periphery	2.230969	.099778	22.36	0.000	2.033702	2.428235
center	2.154901	.1037736	20.77	0.000	1.949735	2.360067

Linear regression

Number of obs	=	145
F(10, 134)	=	1.43
Prob > F	=	0.1734
R-squared	=	0.1060
Root MSE	=	.25567

h6cdd123789	Coef.	Robust Std. Err.	t	P>|t|	[95% Conf.	Interval]
expo	.1297761	.0465553	2.79	0.006	.0376978	.2218543
TRAFFIC						
2	-.0801049	.0564657	-1.42	0.158	-.1917843	.0315745
3	.0033596	.0646956	0.05	0.959	-.1245971	.1313162
4	-.0125283	.0717456	-0.17	0.862	-.1544285	.129372
age						
2	.0367132	.0659438	0.56	0.579	-.0937123	.1671386
3	.0269712	.0523815	0.51	0.607	-.0766303	.1305727
4	.0667932	.057029	1.17	0.244	-.0460003	.1795867
5	.087354	.0907974	0.96	0.338	-.0922273	.2669354
zone						
periphery	.1215099	.0472132	2.57	0.011	.0281305	.2148893
center	.1052891	.0598415	1.76	0.081	-.0130669	.2236451
_cons	.3452336	.0486079	7.10	0.000	.2490955	.4413716

Linear regression

Number of obs	=	148
F(7, 140)	=	1.94
Prob > F	=	0.0672
R-squared	=	0.0902
Root MSE	=	.25356

h6cdd123789	Coef.	Robust Std. Err.	t	P>|t|	[95% Conf.	Interval]
expo	.1182949	.0459509	2.57	0.011	.0274474	.2091424
age						
2	.053562	.0630394	0.85	0.397	-.0710703	.1781944
3	.0305392	.0516049	0.59	0.555	-.0714866	.1325649
4	.0648419	.0573164	1.13	0.260	-.0484756	.1781594
5	.0734894	.0876121	0.84	0.403	-.0997245	.2467033
zone						
periphery	.1168994	.0446852	2.62	0.010	.0285543	.2052445

center	.0840907	.0537949	1.56	0.120	-.0222646	.190446
_cons	.336011	.0491889	6.83	0.000	.2387619	.4332601

```
Linear regression                               Number of obs     =        148
                                                F(3, 144)         =       4.04
                                                Prob > F          =     0.0085
                                                R-squared         =     0.0798
                                                Root MSE          =     .25144
```

h6cdd123789	Coef.	Robust Std. Err.	t	P>|t|	[95% Conf.	Interval]
expo	.1206916	.0447397	2.70	0.008	.0322601	.209123
zone						
periphery	.1300352	.0467064	2.78	0.006	.0377164	.222354
center	.0975377	.0524747	1.86	0.065	-.0061825	.2012579
_cons	.3655159	.040468	9.03	0.000	.285528	.4455039

. margin expo

Predictive margins   Number of obs = 148

Model VCE : Robust

Expression : Linear prediction, predict()

	Margin	Delta-method Std. Err.	t	P>|t|	[95% Conf.	Interval]
expo						
0	.4584333	.0229344	19.99	0.000	.4131018	.5037649
1	.5791249	.0363085	15.95	0.000	.5073585	.6508913

. margin zone

Predictive margins   Number of obs = 148

Model VCE : Robust

Expression : Linear prediction, predict()

	Margin	Delta-method Std. Err.	t	P>|t|	[95% Conf. Interval]

	Coef.	Std. Err.	t	P>|t|	[95% Conf.	Interval]
zone						
rural	.4266772	.0305361	13.97	0.000	.3663202	.4870341
periphery	.5567124	.0331677	16.78	0.000	.4911539	.6222709
center	.5242149	.0394321	13.29	0.000	.4462744	.6021553

Linear regression

Number of obs	=	145
F(10, 134)	=	0.83
Prob > F	=	0.6050
R-squared	=	0.0435
Root MSE	=	1.2036

h7cdd1234678	Coef.	Robust Std. Err.	t	P>|t|	[95% Conf.	Interval]
expo	.2512924	.1930658	1.30	0.195	-.130558	.6331428
TRAFFIC						
2	.358906	.2707635	1.33	0.187	-.1766171	.8944291
3	.4170384	.3012119	1.38	0.168	-.1787063	1.012783
4	-.0348483	.3320532	-0.10	0.917	-.6915916	.621895
age						
2	.145084	.2667661	0.54	0.587	-.3825329	.6727008
3	.1327871	.2954296	0.45	0.654	-.4515212	.7170954
4	.0162031	.2138594	0.08	0.940	-.4067736	.4391797
5	.2318067	.4716812	0.49	0.624	-.7010966	1.16471
zone						
periphery	.1021038	.2235255	0.46	0.649	-.3399906	.5441982
center	.3213292	.2640816	1.22	0.226	-.2009783	.8436366
_cons	1.597088	.2176563	7.34	0.000	1.166602	2.027574

Linear regression

Number of obs	=	145
F(10, 134)	=	1.20
Prob > F	=	0.2945
R-squared	=	0.0520
Root MSE	=	11.882

o8cdd	Coef.	Robust Std. Err.	t	P>|t|	[95% Conf.	Interval]
expo	3.652265	1.994218	1.83	0.069	-.2919502	7.59648
TRAFFIC						
2	-.2335863	2.406508	-0.10	0.923	-4.99324	4.526068

	Coef.	Std. Err.	t	P>|t|	[95% Conf.	Interval]
3	1.447102	2.637126	0.55	0.584	-3.768674	6.662877
4	-1.51593	3.605295	-0.42	0.675	-8.646576	5.614716
age						
2	-2.260881	2.812173	-0.80	0.423	-7.82287	3.301107
3	1.352644	3.670853	0.37	0.713	-5.907664	8.612953
4	-1.819956	3.020541	-0.60	0.548	-7.794059	4.154148
5	-1.593256	4.196764	-0.38	0.705	-9.893723	6.707212
zone						
periphery	3.247646	1.902482	1.71	0.090	-.5151309	7.010424
center	6.329151	2.495293	2.54	0.012	1.393897	11.26441
_cons	14.81102	2.835679	5.22	0.000	9.202545	20.4195

Linear regression

Number of obs	=	148
F(7, 140)	=	1.43
Prob > F	=	0.1978
R-squared	=	0.0532
Root MSE	=	11.809

o8cdd	Coef.	Robust Std. Err.	t	P>|t|	[95% Conf.	Interval]
expo	3.015186	1.790259	1.68	0.094	-.5242519	6.554624
age						
2	-1.811864	2.887466	-0.63	0.531	-7.52054	3.896812
3	2.241167	3.663053	0.61	0.542	-5.000885	9.483218
4	-1.739344	2.962685	-0.59	0.558	-7.596732	4.118044
5	-2.141114	3.802354	-0.56	0.574	-9.658573	5.376345
zone						
periphery	3.447378	1.815573	1.90	0.060	-.1421078	7.036865
center	6.140782	2.682348	2.29	0.024	.8376372	11.44393
_cons	14.86426	2.788068	5.33	0.000	9.352096	20.37641

Linear regression

Number of obs	=	148
F(3, 144)	=	3.23
Prob > F	=	0.0244
R-squared	=	0.0342
Root MSE	=	11.76

o8cdd	Coef.	Robust Std. Err.	t	P>|t|	[95% Conf.	Interval]

expo	2.914335	1.746208	1.67	0.097	-.5371751	6.365846
zone						
periphery	2.865121	1.74528	1.64	0.103	-.5845566	6.314798
center	5.85585	2.448014	2.39	0.018	1.017166	10.69453
_cons	14.74997	1.7094	8.63	0.000	11.37121	18.12872

. margin expo

Predictive margins — Number of obs = 148
Model VCE : Robust

Expression : Linear prediction, predict()

	Delta-method					
	Margin	Std. Err.	t	P>|t|	[95% Conf.	Interval]
expo						
0	18.02449	1.453049	12.40	0.000	15.15243	20.89655
1	20.93883	1.13712	18.41	0.000	18.69122	23.18643

. margin zone

Predictive margins — Number of obs = 148
Model VCE : Robust

Expression : Linear prediction, predict()

	Margin	Delta-method Std. Err.	t	P>|t|	[95% Conf.	Interval]
zone						
rural	16.22682	1.294409	12.54	0.000	13.66833	18.78532
periphery	19.09194	1.159384	16.47	0.000	16.80034	21.38355
center	22.08267	2.175073	10.15	0.000	17.78348	26.38187

```
Linear regression                               Number of obs     =        148
                                                F(7, 140)         =      10.01
                                                Prob > F          =     0.0000
                                                R-squared         =     0.2898
                                                Root MSE          =     .20943
```

t4cdd2378	Coef.	Robust Std. Err.	t	P>|t|	[95% Conf.	Interval]
expo	.2222409	.0374569	5.93	0.000	.1481866	.2962951

age						
2	.053259	.0548866	0.97	0.334	-.0552547	.1617727
3	.001222	.057958	0.02	0.983	-.1133642	.1158081
4	.1091835	.046896	2.33	0.021	.0164676	.2018993
5	.0815258	.0707301	1.15	0.251	-.0583114	.221363
zone						
periphery	.0894393	.0470333	1.90	0.059	-.0035481	.1824267
center	.0313295	.0476966	0.66	0.512	-.0629692	.1256282
_cons	.3264603	.0477733	6.83	0.000	.2320099	.4209107

. margin expo

Predictive margins   Number of obs = 148
Model VCE : Robust

Expression : Linear prediction, predict()

	Delta-method					
	Margin	Std. Err.	t	P>|t|	[95% Conf.	Interval]
expo						
0	.4255536	.0233683	18.21	0.000	.3793532	.471754
1	.6477945	.0273007	23.73	0.000	.5938195	.7017695

Linear regression

Number of obs	=	148
F(7, 140)	=	4.16
Prob > F	=	0.0003
R-squared	=	0.1593
Root MSE	=	.41898

p5cdd12378	Coef.	Robust Std. Err.	t	P>|t|	[95% Conf. Interval]	
expo	.2154195	.0702873	3.06	0.003	.0764578	.3543812
age						
2	.0914715	.1099744	0.83	0.407	-.1259538	.3088969
3	.1530431	.1026641	1.49	0.138	-.0499294	.3560156
4	.2896353	.0929372	3.12	0.002	.1058935	.4733772
5	.3306251	.1216434	2.72	0.007	.0901297	.5711206
zone						
periphery	.1613574	.0862788	1.87	0.064	-.0092204	.3319352
center	.1087397	.0874193	1.24	0.216	-.0640929	.2815723
_cons	.8006694	.0979887	8.17	0.000	.6069405	.9943984

. margin expo

Predictive margins Number of obs = 148
Model VCE : Robust

Expression : Linear prediction, predict()

	Delta-method Margin	Std. Err.	t	P>|t|	[95% Conf.	Interval]
expo						
0	1.075294	.0512624	20.98	0.000	.9739456	1.176643
1	1.290714	.0471123	27.40	0.000	1.19757	1.383857

Linear regression

Number of obs = 148
F(7, 140) = 4.00
Prob > F = 0.0005
R-squared = 0.1368
Root MSE = .2026

h6cdd123478	Coef.	Robust Std. Err.	t	P>|t|	[95% Conf.	Interval]
expo	.1113834	.0335561	3.32	0.001	.0450413	.1777256
age						
2	.0824386	.0652509	1.26	0.209	-.046566	.2114432
3	.0541003	.0418331	1.29	0.198	-.028606	.1368065
4	.0929117	.0389343	2.39	0.018	.0159364	.1698869
5	.1179613	.0645753	1.83	0.070	-.0097074	.2456301
zone						
periphery	.1025411	.0386892	2.65	0.009	.0260505	.1790317
center	.1056331	.039644	2.66	0.009	.0272548	.1840115
_cons	.3373224	.0383966	8.79	0.000	.2614102	.4132346

. margin expo

Predictive margins Number of obs = 148
Model VCE : Robust

Expression : Linear prediction, predict()

	Delta-method Margin	Std. Err.	t	P>|t|	[95% Conf. Interval]

expo						
0	.4840963	.0200601	24.13	0.000	.4444364	.5237561
1	.5954797	.0266645	22.33	0.000	.5427625	.6481968

Linear regression

Number of obs	=	148
F(7, 140)	=	5.82
Prob > F	=	0.0000
R-squared	=	0.2282
Root MSE	=	.74639

h6cdd123678	Coef.	Robust Std. Err.	t	P>|t|	[95% Conf. Interval]	
expo	.3057105	.1222387	2.50	0.014	.0640381	.5473829
age						
2	.3182974	.2103488	1.51	0.132	-.0975735	.7341684
3	.393293	.1815896	2.17	0.032	.0342807	.7523053
4	.7776884	.1791266	4.34	0.000	.4235455	1.131831
5	.8924001	.2530633	3.53	0.001	.3920804	1.39272
zone						
periphery	.3552005	.14128	2.51	0.013	.0758824	.6345186
center	.2791326	.1409497	1.98	0.050	.0004676	.5577976
_cons	1.273057	.1724555	7.38	0.000	.9321035	1.614011

```
. margin expo
```

Predictive margins — Number of obs = 148
Model VCE : Robust

Expression : Linear prediction, predict()

	Margin	Delta-method Std. Err.	t	P>|t|	[95% Conf.	Interval]
expo						
0	1.978863	.087337	22.66	0.000	1.806193	2.151533
1	2.284573	.0858706	26.60	0.000	2.114803	2.454344

Linear regression

Number of obs	=	145
F(10, 134)	=	6.55
Prob > F	=	0.0000

R-squared	=	0.3389
Root MSE	=	1.4204

p5cdf23478	Coef.	Robust Std. Err.	t	P>|t|	[95% Conf.	Interval]
expo	1.767109	.2497624	7.08	0.000	1.273122	2.261095
TRAFFIC						
2	-.2852144	.3111258	-0.92	0.361	-.900567	.3301382
3	.1241631	.5428666	0.23	0.819	-.9495324	1.197859
4	-.4027194	.3220047	-1.25	0.213	-1.039589	.2341498
age						
2	.089711	.4362974	0.21	0.837	-.7732092	.9526312
3	.1267203	.3248141	0.39	0.697	-.5157053	.769146
4	.8513626	.3038708	2.80	0.006	.2503591	1.452366
5	1.183493	.4306951	2.75	0.007	.331653	2.035333
zone						
periphery	.7465401	.3079292	2.42	0.017	.1375099	1.35557
center	.8713457	.3269665	2.66	0.009	.2246629	1.518029
_cons	2.936764	.3929332	7.47	0.000	2.159611	3.713918

Linear regression

Number of obs	=	148
F(7, 140)	=	8.82
Prob > F	=	0.0000
R-squared	=	0.3223
Root MSE	=	1.4077

p5cdf23478	Coef.	Robust Std. Err.	t	P>|t|	[95% Conf.	Interval]
expo	1.655919	.2473694	6.69	0.000	1.166857	2.144982
age						
2	.1987275	.4045304	0.49	0.624	-.6010509	.9985059
3	.2131396	.3309884	0.64	0.521	-.4412423	.8675215
4	.8515468	.3099976	2.75	0.007	.2386648	1.464429
5	1.070022	.4007508	2.67	0.008	.2777158	1.862327
zone						
periphery	.7304638	.3103742	2.35	0.020	.1168374	1.34409
center	.714152	.3134802	2.28	0.024	.0943848	1.333919
_cons	2.905975	.3815487	7.62	0.000	2.151633	3.660317

. margin expo

Predictive margins Number of obs = 148
Model VCE : Robust

Expression : Linear prediction, predict()

	Margin	Delta-method Std. Err.	t	P>|t|	[95% Conf.	Interval]
expo						
0	3.912879	.1638507	23.88	0.000	3.588937	4.23682
1	5.568798	.174568	31.90	0.000	5.223668	5.913928

. margin zone

Predictive margins Number of obs = 148
Model VCE : Robust

Expression : Linear prediction, predict()

	Margin	Delta-method Std. Err.	t	P>|t|	[95% Conf.	Interval]
zone						
rural	4.175031	.2471471	16.89	0.000	3.686408	4.663654
periphery	4.905495	.1850641	26.51	0.000	4.539613	5.271376
center	4.889183	.1771305	27.60	0.000	4.538986	5.239379

. margin age

Predictive margins Number of obs = 148
Model VCE : Robust

Expression : Linear prediction, predict()

	Margin	Delta-method Std. Err.	t	P>|t|	[95% Conf.	Interval]
age						
1	4.32212	.2286144	18.91	0.000	3.870137	4.774103
2	4.520847	.3362384	13.45	0.000	3.856086	5.185608
3	4.535259	.2430272	18.66	0.000	4.054781	5.015737
4	5.173666	.2160533	23.95	0.000	4.746517	5.600815
5	5.392141	.3233482	16.68	0.000	4.752865	6.031418

Linear regression

Number of obs	=	145
F(10, 134)	=	5.42
Prob > F	=	0.0000
R-squared	=	0.2627
Root MSE	=	.5328

h6cdf123478	Coef.	Robust Std. Err.	t	P>|t|	[95% Conf.	Interval]
expo	.6279993	.0937942	6.70	0.000	.4424907	.813508
TRAFFIC						
2	-.0776545	.1219387	-0.64	0.525	-.3188281	.163519
3	.0453607	.1636949	0.28	0.782	-.2783993	.3691208
4	-.1379021	.1426585	-0.97	0.335	-.4200558	.1442516
age						
2	-.076705	.1486953	-0.52	0.607	-.3707984	.2173883
3	-.1085324	.135827	-0.80	0.426	-.3771745	.1601097
4	.006536	.131166	0.05	0.960	-.2528874	.2659595
5	-.0799328	.1846598	-0.43	0.666	-.4451577	.285292
zone						
periphery	.2386001	.1030257	2.32	0.022	.0348333	.4423669
center	.3484804	.1310616	2.66	0.009	.0892634	.6076973
_cons	.9776077	.1229609	7.95	0.000	.7344124	1.220803

Linear regression

Number of obs	=	148
F(7, 140)	=	7.61
Prob > F	=	0.0000
R-squared	=	0.2591
Root MSE	=	.52456

h6cdf123478	Coef.	Robust Std. Err.	t	P>|t|	[95% Conf.	Interval]
expo	.6009148	.0891676	6.74	0.000	.4246257	.777204
age						
2	-.0524571	.1397746	-0.38	0.708	-.3287989	.2238847
3	-.0923784	.1371142	-0.67	0.502	-.3634607	.1787038
4	.0074346	.1280405	0.06	0.954	-.2457084	.2605775
5	-.1223085	.1691791	-0.72	0.471	-.4567846	.2121676
zone						
periphery	.2328891	.1017557	2.29	0.024	.0317126	.4340655
center	.2903225	.1253525	2.32	0.022	.0424939	.5381511

_cons	.9679227	.1200766	8.06	0.000	.7305248	1.205321

```
Linear regression                               Number of obs     =        148
                                                F(3, 144)         =      16.81
                                                Prob > F          =     0.0000
                                                R-squared         =     0.2524
                                                Root MSE          =     .51955

------------------------------------------------------------------------------
             |               Robust
 h6cdf123478 |      Coef.   Std. Err.      t    P>|t|     [95% Conf. Interval]
-------------+----------------------------------------------------------------
        expo |   .6127285   .0867143     7.07   0.000     .4413311    .7841258
             |
        zone |
   periphery |    .216788    .100631     2.15   0.033     .0178833    .4156928
      center |   .2772584   .1170102     2.37   0.019     .0459789    .5085378
             |
       _cons |   .9310901   .0957886     9.72   0.000     .7417566    1.120423
------------------------------------------------------------------------------

. margin expo

Predictive margins                              Number of obs     =        148
Model VCE    : Robust

Expression   : Linear prediction, predict()

------------------------------------------------------------------------------
             |            Delta-method
             |     Margin   Std. Err.      t    P>|t|     [95% Conf. Interval]
-------------+----------------------------------------------------------------
        expo |
          0  |   1.123955   .0560113    20.07   0.000     1.013245    1.234666
          1  |   1.736684   .0652253    26.63   0.000     1.607761    1.865606
------------------------------------------------------------------------------
```

. margin zone

Predictive margins Number of obs = 148

Model VCE : Robust

Expression : Linear prediction, predict()

	Margin	Delta-method Std. Err.	t	P>|t|	[95% Conf.	Interval]
zone						
rural	1.241594	.0781889	15.88	0.000	1.087048	1.396141
periphery	1.458382	.0606158	24.06	0.000	1.338571	1.578194

center	1.518853	.0846134	17.95	0.000	1.351608	1.686097

Linear regression

Number of obs	=	148
F(7, 140)	=	9.62
Prob > F	=	0.0000
R-squared	=	0.2861
Root MSE	=	.52744

h6cdf123678	Coef.	Robust Std. Err.	t	P>|t|	[95% Conf.	Interval]
expo	.6394998	.0903654	7.08	0.000	.4608426	.818157
age						
2	-.035649	.1486527	-0.24	0.811	-.3295434	.2582455
3	-.0608106	.1361594	-0.45	0.656	-.3300049	.2083838
4	.0600898	.1268323	0.47	0.636	-.1906645	.3108441
5	-.0142836	.1712153	-0.08	0.934	-.3527855	.3242183
zone						
periphery	.3012255	.0989712	3.04	0.003	.1055541	.4968968
center	.3508734	.1196179	2.93	0.004	.1143825	.5873644
_cons	.9117932	.1191446	7.65	0.000	.6762379	1.147348

Linear regression

Number of obs	=	148
F(3, 144)	=	19.94
Prob > F	=	0.0000
R-squared	=	0.2810
Root MSE	=	.52191

h6cdf123678	Coef.	Robust Std. Err.	t	P>|t|	[95% Conf.	Interval]
expo	.651352	.0862506	7.55	0.000	.4808712	.8218328
zone						
periphery	.3025735	.0991622	3.05	0.003	.1065719	.4985751
center	.3562808	.1105754	3.22	0.002	.1377203	.5748413
_cons	.8973869	.0925921	9.69	0.000	.7143717	1.080402

. margin expo

Predictive margins   Number of obs = 148

Model VCE : Robust

Expression : Linear prediction, predict()

	Margin	Delta-method Std. Err.	t	P>|t|	[95% Conf.	Interval]
expo						
0	1.156409	.0578036	20.01	0.000	1.042156	1.270663
1	1.807761	.0636318	28.41	0.000	1.681988	1.933535

. margin zone

Predictive margins Number of obs = 148
Model VCE : Robust

Expression : Linear prediction, predict()

	Margin	Delta-method Std. Err.	t	P>|t|	[95% Conf.	Interval]
zone						
rural	1.227464	.0735552	16.69	0.000	1.082077	1.372851
periphery	1.530037	.0641402	23.85	0.000	1.403259	1.656815
center	1.583745	.080839	19.59	0.000	1.42396	1.743529

```
Linear regression                               Number of obs     =        148
                                                F(7, 140)         =       4.62
                                                Prob > F          =     0.0001
                                                R-squared         =     0.1731
                                                Root MSE          =     .43875
```

h6cdf234678	Coef.	Robust Std. Err.	t	P>|t|	[95% Conf.	Interval]
expo	.3711879	.074864	4.96	0.000	.2231777	.5191981
age						
2	.0778526	.1442621	0.54	0.590	-.2073614	.3630665
3	-.041889	.1016173	-0.41	0.681	-.242792	.1590139
4	-.0566149	.0881503	-0.64	0.522	-.2308928	.117663
5	-.11029	.1130726	-0.98	0.331	-.3338405	.1132605
zone						
periphery	.2258477	.084984	2.66	0.009	.0578298	.3938655
center	.2305219	.0985595	2.34	0.021	.0356644	.4253794
_cons	.4301773	.0835119	5.15	0.000	.2650699	.5952848

Linear regression

Number of obs	= 148
F(3, 144)	= 10.10
Prob > F	= 0.0000
R-squared	= 0.1590
Root MSE	= .43629

h6cdf234678	Coef.	Robust Std. Err.	t	P>|t|	[95% Conf.	Interval]
expo	.3674065	.0694795	5.29	0.000	.2300751	.5047379
zone						
periphery	.211549	.0851946	2.48	0.014	.0431555	.3799424
center	.2055295	.0865778	2.37	0.019	.0344019	.3766571
_cons	.4228154	.0714357	5.92	0.000	.2816174	.5640135

. margin expo

Predictive margins   Number of obs = 148
Model VCE   : Robust

Expression   : Linear prediction, predict()

	Margin	Delta-method Std. Err.	t	P>|t|	[95% Conf.	Interval]
expo						
0	.5894899	.0402356	14.65	0.000	.5099613	.6690185
1	.9568965	.0578215	16.55	0.000	.8426079	1.071185

. margin zone

Predictive margins   Number of obs = 148
Model VCE   : Robust

Expression   : Linear prediction, predict()

	Margin	Delta-method Std. Err.	t	P>|t|	[95% Conf.	Interval]
zone						
rural	.6090012	.0591555	10.29	0.000	.492076	.7259264
periphery	.8205502	.0553473	14.83	0.000	.7111521	.9299482
center	.8145307	.0631016	12.91	0.000	.6898056	.9392557

.

```
Linear regression                               Number of obs     =        148
                                                F(7, 140)         =       4.76
                                                Prob > F          =     0.0001
                                                R-squared         =     0.1113
                                                Root MSE          =     .75523

------------------------------------------------------------------------------
             |               Robust
 h7cdf1234678 |      Coef.   Std. Err.      t    P>|t|     [95% Conf. Interval]
-------------+----------------------------------------------------------------
        expo |   .3819986   .1049301     3.64   0.000     .1745461     .589451
             |
         age |
          2  |   .0817993   .2504965     0.33   0.744    -.4134458    .5770443
          3  |  -.0131248   .1667667    -0.08   0.937    -.3428315     .316582
          4  |  -.1180315   .1520195    -0.78   0.439    -.4185823    .1825193
          5  |  -.2647718   .1576851    -1.68   0.095    -.5765237    .0469801
             |
        zone |
   periphery |   .0944889   .0944649     1.00   0.319    -.0922732    .2812511
      center |   .5320418   .1924044     2.77   0.006      .151648    .9124357
             |
       _cons |   .4262376   .1255887     3.39   0.001      .177942    .6745332
------------------------------------------------------------------------------

. margin expo

Predictive margins                              Number of obs     =        148
Model VCE    : Robust

Expression   : Linear prediction, predict()

------------------------------------------------------------------------------
             |            Delta-method
             |     Margin   Std. Err.      t    P>|t|     [95% Conf. Interval]
-------------+----------------------------------------------------------------
        expo |
          0  |   .5939196    .085967     6.91   0.000     .4239584    .7638809
          1  |   .9759182   .0764364    12.77   0.000     .8247993    1.127037
------------------------------------------------------------------------------
```

. margin zone

Predictive margins Number of obs = 148

Model VCE : Robust

Expression : Linear prediction, predict()

```
--------------------------------------------------------------------------------------
             |            Delta-method
             |     Margin   Std. Err.                                    t    P>|
             +----------------------------------------------------------------
        zone |
```

	Margin	Std. Err.	t	P>|t|	[95% Conf.	Interval]
rural	.5672987	.0798615	7.10	0.000	.4094083	.7251892
periphery	.6617877	.0481696	13.74	0.000	.5665539	.7570215
center	1.099341	.1711797	6.42	0.000	.7609091	1.437772

. margin age

Predictive margins   Number of obs = 148
Model VCE : Robust

Expression : Linear prediction, predict()

	Margin	Delta-method Std. Err.	t	P>|t|	[95% Conf.	Interval]
age						
1	.8400192	.1082822	7.76	0.000	.6259395	1.054099
2	.9218185	.2361179	3.90	0.000	.4550006	1.388636
3	.8268944	.1268898	6.52	0.000	.5760264	1.077762
4	.7219877	.1074279	6.72	0.000	.5095969	.9343784
5	.5752474	.1077279	5.34	0.000	.3622635	.7882313

Linear regression   Number of obs = 145
F(10, 134) = 2.04
Prob > F = 0.0336
R-squared = 0.1820
Root MSE = 8.2239

p5cb126	Coef.	Robust Std. Err.	t	P>|t|	[95% Conf.	Interval]
expo	4.634896	1.463739	3.17	0.002	1.739875	7.529917
TRAFFIC						
2	-2.418639	1.646657	-1.47	0.144	-5.675439	.8381614
3	5.46365	3.148491	1.74	0.085	-.763517	11.69082
4	-1.766922	2.005392	-0.88	0.380	-5.733238	2.199393
age						
2	.5630386	1.861833	0.30	0.763	-3.119343	4.245421
3	1.403708	1.914035	0.73	0.465	-2.381921	5.189336
4	4.126947	1.924354	2.14	0.034	.3209104	7.932984
5	5.992485	2.660962	2.25	0.026	.7295658	11.2554
zone						
periphery	3.576616	1.94929	1.83	0.069	-.2787401	7.431973
center	4.734853	2.186058	2.17	0.032	.4112117	9.058494

_cons	11.47003	2.103611	5.45	0.000	7.309451	15.6306

. margin expo

Predictive margins Number of obs = 145
Model VCE : Robust

Expression : Linear prediction, predict()

	Delta-method					
	Margin	Std. Err.	t	P>|t|	[95% Conf.	Interval]
expo						
0	16.47919	.9103993	18.10	0.000	14.67858	18.2798
1	21.11409	1.078208	19.58	0.000	18.98158	23.24659

. margin age

Predictive margins Number of obs = 145
Model VCE : Robust

Expression : Linear prediction, predict()

	Delta-method					
	Margin	Std. Err.	t	P>|t|	[95% Conf.	Interval]
age						
1	16.68628	1.327307	12.57	0.000	14.0611	19.31146
2	17.24932	1.30017	13.27	0.000	14.67781	19.82083
3	18.08999	1.443321	12.53	0.000	15.23535	20.94462
4	20.81323	1.415147	14.71	0.000	18.01431	23.61214
5	22.67876	2.251288	10.07	0.000	18.22611	27.13142

. margin zone

Predictive margins Number of obs = 145
Model VCE : Robust

Expression : Linear prediction, predict()

	Delta-method Std.					
	Margin	Err.	t	P>|t|	[95% Conf.	Interval]
zone						
rurale	15.6645	1.688289	9.28	0.000	12.32535	19.00364
periferia	19.24111	1.025019	18.77	0.000	17.2138	21.26842
centro	20.39935	1.26183	16.17	0.000	17.90367	22.89503

Linear regression

Number of obs	=	145
F(10, 134)	=	5.86
Prob > F	=	0.0000
R-squared	=	0.2543
Root MSE	=	5.6301

PCB169	Coef.	Robust Std. Err.	t	P>|t|	[95% Conf.	Interval]
expo	1.808077	1.071411	1.69	0.094	-.3109879	3.927142
TRAFFIC						
2	-1.849383	1.206101	-1.53	0.128	-4.23484	.5360749
3	-.0162383	1.716541	-0.01	0.992	-3.411257	3.378781
4	-1.751778	1.171835	-1.49	0.137	-4.069463	.565907
age						
2	2.535862	1.540759	1.65	0.102	-.5114922	5.583215
3	3.482177	1.61329	2.16	0.033	.2913703	6.672983
4	6.295288	1.233107	5.11	0.000	3.856416	8.73416
5	7.874413	1.487889	5.29	0.000	4.931628	10.8172
zone						
periphery	3.027462	1.413378	2.14	0.034	.2320464	5.822878
center	2.899128	1.25756	2.31	0.023	.4118932	5.386363
_cons	7.022518	1.642003	4.28	0.000	3.774921	10.27011

Linear regression

Number of obs	=	148
F(7, 140)	=	7.15
Prob > F	=	0.0000
R-squared	=	0.2361
Root MSE	=	5.631

PCB169	Coef.	Robust Std. Err.	t	P>|t|	[95% Conf.	Interval]
expo	1.389049	1.000396	1.39	0.167	-.5887885	3.366886
age						
2	2.78841	1.481089	1.88	0.062	-.1397832	5.716603
3	3.907124	1.592517	2.45	0.015	.7586324	7.055615
4	6.216704	1.233065	5.04	0.000	3.778869	8.65454
5	7.637646	1.419301	5.38	0.000	4.831612	10.44368
zone						

periphery	2.702699	1.348876	2.00	0.047	.0358982	5.369501
center	2.24985	1.219222	1.85	0.067	-.1606174	4.660317
_cons	6.767371	1.642928	4.12	0.000	3.519213	10.01553

. margin age

Predictive margins   Number of obs = 148
Model VCE : Robust

Expression : Linear prediction, predict()

	Margin	Delta-method Std. Err.	t	P>|t|	[95% Conf.	Interval]
age						
1	9.476206	.9719428	9.75	0.000	7.554623	11.39779
2	12.26462	1.114853	11.00	0.000	10.06049	14.46874
3	13.38333	1.29655	10.32	0.000	10.81998	15.94668
4	15.69291	.7790592	20.14	0.000	14.15267	17.23315
5	17.11385	1.009622	16.95	0.000	15.11777	19.10993

. margin zone

Predictive margins   Number of obs = 148
Model VCE : Robust

Expression : Linear prediction, predict()

	Margin	Delta-method Std. Err.	t	P>|t|	[95% Conf.	Interval]
zone						
rural	11.32176	.9934276	11.40	0.000	9.357704	13.28582
periphery	14.02446	.8220124	17.06	0.000	12.3993	15.64963
center	13.57161	.6063383	22.38	0.000	12.37285	14.77038

```
Linear regression                               Number of obs     =        145
                                                F(10, 134)        =       3.59
                                                Prob > F          =     0.0003
                                                R-squared         =     0.2026
                                                Root MSE          =     396.58
```

p5cb105	Coef.	Robust Std. Err.	t	P>|t|	[95% Conf.	Interval]

expo	303.6628	69.8259	4.35	0.000	165.5594	441.7663
TRAFFIC						
2	-129.8279	80.80311	-1.61	0.110	-289.6424	29.98658
3	54.93936	121.9484	0.45	0.653	-186.2534	296.1321
4	-119.7197	106.4115	-1.13	0.263	-330.1831	90.74374
age						
2	4.34907	81.14212	0.05	0.957	-156.1359	164.834
3	43.43168	85.60781	0.51	0.613	-125.8856	212.749
4	220.7846	86.94905	2.54	0.012	48.81449	392.7546
5	254.374	135.662	1.88	0.063	-13.94187	522.6899
zone						
periphery	169.843	78.15793	2.17	0.032	15.26025	324.4258
center	313.5122	107.7739	2.91	0.004	100.3542	526.6702
_cons	524.6487	81.9941	6.40	0.000	362.4787	686.8188

```
Linear regression                               Number of obs     =        148
                                                F(7, 140)         =       4.31
                                                Prob > F          =     0.0002
                                                R-squared         =     0.1631
                                                Root MSE          =     409.38
```

p5cb105	Coef.	Robust Std. Err.	t	P>|t|	[95% Conf.	Interval]
expo	266.5043	71.05545	3.75	0.000	126.0239	406.9847
age						
2	31.39221	81.04211	0.39	0.699	-128.8324	191.6168
3	93.40184	93.47384	1.00	0.319	-91.40097	278.2046
4	219.5991	88.0224	2.49	0.014	45.57409	393.6241
5	180.5686	141.3952	1.28	0.204	-98.97725	460.1144
zone						
periphery	167.3898	75.75733	2.21	0.029	17.61345	317.1661
center	263.1919	94.59595	2.78	0.006	76.17059	450.2132
_cons	506.9814	80.63316	6.29	0.000	347.5653	666.3975

. margin expo

Predictive margins — Number of obs = 148

Model VCE : Robust

Expression : Linear prediction, predict()

	Margin	Delta-method Std. Err.	t	P>|t|	[95% Conf.	Interval]
expo						
0	775.7377	40.80435	19.01	0.000	695.0653	856.4101
1	1042.242	55.71597	18.71	0.000	932.0885	1152.395

. margin age

Predictive margins — Number of obs = 148
Model VCE : Robust

Expression : Linear prediction, predict()

	Margin	Delta-method Std. Err.	t	P>|t|	[95% Conf.	Interval]
age						
1	807.2119	54.34637	14.85	0.000	699.7663	914.6576
2	838.6041	61.22966	13.70	0.000	717.5498	959.6585
3	900.6138	79.53935	11.32	0.000	743.3602	1057.867
4	1026.811	68.01158	15.10	0.000	892.3485	1161.274
5	987.7805	129.6462	7.62	0.000	731.463	1244.098

. margin zone

Predictive margins — Number of obs = 148
Model VCE : Robust

Expression : Linear prediction, predict()

	Margin	Delta-method Std. Err.	t	P>|t|	[95% Conf.	Interval]
zone						
rural	745.6129	61.41304	12.14	0.000	624.196	867.0298
periphery	913.0026	44.11786	20.69	0.000	825.7792	1000.226
center	1008.805	69.90011	14.43	0.000	870.6085	1147.001

Linear regression

Number of obs	=	145
F(10, 134)	=	4.37
Prob > F	=	0.0000
R-squared	=	0.2669
Root MSE	=	97.507

p5cb114	Coef.	Robust Std. Err.	t	P>|t|	[95% Conf.	Interval]
expo	51.00938	18.89639	2.70	0.008	13.63561	88.38315
TRAFFIC						
2	-22.88094	21.81424	-1.05	0.296	-66.02571	20.26384
3	-4.884534	29.11851	-0.17	0.867	-62.47588	52.70681
4	-15.8641	22.88661	-0.69	0.489	-61.12982	29.40162
age						
2	28.75892	24.30269	1.18	0.239	-19.30757	76.82542
3	44.69063	23.9616	1.87	0.064	-2.701232	92.08249
4	108.6784	24.16356	4.50	0.000	60.8871	156.4697
5	121.5131	30.50111	3.98	0.000	61.1872	181.8389
zone						
periphery	47.05338	20.49887	2.30	0.023	6.510177	87.59658
center	55.82181	24.96266	2.24	0.027	6.450012	105.1936
_cons	124.4148	25.6268	4.85	0.000	73.72941	175.1001

```
Linear regression                               Number of obs     =        148
                                                F(7, 140)         =       5.64
                                                Prob > F          =     0.0000
                                                R-squared         =     0.2480
                                                Root MSE          =     98.302
```

p5cb114	Coef.	Robust Std. Err.	t	P>|t|	[95% Conf.	Interval]
1.expo	45.74666	17.4259	2.63	0.010	11.29472	80.19859
age						
2	31.65119	23.34257	1.36	0.177	-14.49833	77.80071
3	53.44102	24.97886	2.14	0.034	4.056481	102.8256
4	107.2788	23.95724	4.48	0.000	59.91401	154.6435
5	117.1391	29.77162	3.93	0.000	58.27898	175.9992
zone						
periphery	43.48526	20.80543	2.09	0.038	2.351807	84.61872
center	51.54643	23.52853	2.19	0.030	5.029248	98.0636
_cons	120.8675	25.32766	4.77	0.000	70.79339	170.9417

. margin expo

Predictive margins   Number of obs = 148

Model VCE : Robust

Expression : Linear prediction, predict()

	Margin	Delta-method Std. Err.	t	P>|t|	[95% Conf.	Interval]
expo						
0	216.9662	9.478574	22.89	0.000	198.2266	235.7059
1	262.7129	13.82662	19.00	0.000	235.3769	290.0489

. margin age

Predictive margins   Number of obs = 148
Model VCE : Robust

Expression : Linear prediction, predict()

	Margin	Delta-method Std. Err.	t	P>|t|	[95% Conf.	Interval]
age						
1	181.3895	17.99004	10.08	0.000	145.8223	216.9568
2	213.0407	15.01413	14.19	0.000	183.357	242.7245
3	234.8306	17.97894	13.06	0.000	199.2852	270.3759
4	288.6683	15.88952	18.17	0.000	257.2539	320.0827
5	298.5286	23.74822	12.57	0.000	251.5771	345.4801

. margin zone

Predictive margins   Number of obs = 148
Model VCE : Robust

Expression : Linear prediction, predict()

	Margin	Delta-method Std. Err.	t	P>|t|	[95% Conf.	Interval]
zone						
rural	202.8091	16.60713	12.21	0.000	169.9759	235.6423
periphery	246.2943	11.73419	20.99	0.000	223.0952	269.4935
center	254.3555	15.6445	16.26	0.000	223.4255	285.2855

Linear regression

Number of obs	=	145
F(10, 134)	=	4.24
Prob > F	=	0.0000
R-squared	=	0.2430
Root MSE	=	2038.5

p5cb118	Coef.	Robust Std. Err.	t	P>|t|	[95% Conf.	Interval]
expo	1604.314	348.0094	4.61	0.000	916.0119	2292.616
TRAFFIC						
2	-677.5059	417.4273	-1.62	0.107	-1503.104	148.0925
3	244.0835	659.609	0.37	0.712	-1060.508	1548.675
4	-386.4939	524.8842	-0.74	0.463	-1424.623	651.6355
age						
2	437.8521	452.4048	0.97	0.335	-456.9259	1332.63
3	457.9521	410.7716	1.11	0.267	-354.4826	1270.387
4	1580.341	459.6565	3.44	0.001	671.2211	2489.462
5	1917.184	730.1728	2.63	0.010	473.0294	3361.339
zone						
periphery	928.4445	444.2989	2.09	0.039	49.69861	1807.19
center	1312.491	521.3962	2.52	0.013	281.26	2343.722
_cons	2774.618	427.6816	6.49	0.000	1928.738	3620.497

```
Linear regression                               Number of obs     =        148
                                                F(7, 140)         =       5.56
                                                Prob > F          =     0.0000
                                                R-squared         =     0.2017
                                                Root MSE          =       2103
```

p5cb118	Coef.	Robust Std. Err.	t	P>|t|	[95% Conf.	Interval]
expo	1447.537	354.794	4.08	0.000	746.09	2148.984
age						
2	545.0128	453.5561	1.20	0.232	-351.6919	1441.718
3	697.1655	453.36	1.54	0.126	-199.1516	1593.483
4	1570.497	461.8448	3.40	0.001	657.4053	2483.589
5	1566.479	772.5037	2.03	0.044	39.19814	3093.761
zone						
periphery	920.3223	433.744	2.12	0.036	62.78709	1777.857
center	1117.146	467.5039	2.39	0.018	192.8652	2041.426
_cons	2682.434	422.7473	6.35	0.000	1846.64	3518.228

. margin expo

Predictive margins   Number of obs = 148
Model VCE : Robust

Expression : Linear prediction, predict()

	Margin	Delta-method Std. Err.	t	P>|t|	[95% Conf.	Interval]
expo						
0	4317.897	206.4665	20.91	0.000	3909.701	4726.092
1	5765.434	282.0754	20.44	0.000	5207.756	6323.112

. margin age

Predictive margins   Number of obs = 148
Model VCE : Robust

Expression : Linear prediction, predict()

	Margin	Delta-method Std. Err.	t	P>|t|	[95% Conf.	Interval]
age						
1	4214.918	291.907	14.44	0.000	3637.802	4792.034
2	4759.931	343.5026	13.86	0.000	4080.808	5439.054
3	4912.084	364.1253	13.49	0.000	4192.188	5631.979
4	5785.415	349.407	16.56	0.000	5094.619	6476.212
5	5781.397	706.5412	8.18	0.000	4384.528	7178.267

. margin zone

Predictive margins   Number of obs = 148
Model VCE : Robust

Expression : Linear prediction, predict()

	Margin	Delta-method Std. Err.	t	P>|t|	[95% Conf.	Interval]
zone						
rural	4252.511	352.8314	12.05	0.000	3554.944	4950.078
periphery	5172.833	247.2728	20.92	0.000	4683.962	5661.705
center	5369.657	306.5259	17.52	0.000	4763.638	5975.675

Linear regression   Number of obs = 145
F(10, 134) = 3.06

Prob > F	=	0.0016
R-squared	=	0.2135
Root MSE	=	27.566

p5cb123	Coef.	Robust Std. Err.	t	P>|t|	[95% Conf.	Interval]
expo	22.36085	4.904134	4.56	0.000	12.66133	32.06038
TRAFFIC						
2	-6.783911	6.087204	-1.11	0.267	-18.82334	5.255518
3	6.465007	8.473343	0.76	0.447	-10.29379	23.2238
4	-6.071534	7.108702	-0.85	0.395	-20.13131	7.988239
age						
2	.152739	5.35126	0.03	0.977	-10.43112	10.7366
3	5.906451	5.895996	1.00	0.318	-5.754802	17.5677
4	16.54329	6.759477	2.45	0.016	3.174217	29.91235
5	18.96094	8.759258	2.16	0.032	1.636659	36.28523
zone						
periphery	11.53384	6.492902	1.78	0.078	-1.307989	24.37567
center	17.67692	8.109197	2.18	0.031	1.638342	33.7155
_cons	36.37026	6.589949	5.52	0.000	23.33649	49.40403

Linear regression

Number of obs	=	148
F(7, 140)	=	4.12
Prob > F	=	0.0004
R-squared	=	0.1831
Root MSE	=	28.042

p5cb123	Coef.	Robust Std. Err.	t	P>|t|	[95% Conf.	Interval]
expo	19.91557	4.818711	4.13	0.000	10.38872	29.44242
age						
2	1.79145	5.394141	0.33	0.740	-8.873056	12.45596
3	9.099792	6.474285	1.41	0.162	-3.700217	21.8998
4	16.71711	6.693238	2.50	0.014	3.48422	29.95
5	14.75404	8.967988	1.65	0.102	-2.97616	32.48423
zone						
periphery	11.92708	6.21426	1.92	0.057	-.3588455	24.21301
center	15.11109	7.155541	2.11	0.036	.9642029	29.25798
_cons	35.70325	6.533294	5.46	0.000	22.78658	48.61992

. margin expo

Predictive margins Number of obs = 148
Model VCE : Robust

Expression : Linear prediction, predict()

	Margin	Delta-method Std. Err.	t	P>|t|	[95% Conf.	Interval]
expo						
0	54.61035	2.775108	19.68	0.000	49.12382	60.09689
1	74.52592	3.800306	19.61	0.000	67.01251	82.03933

. margin age

Predictive margins Number of obs = 148
Model VCE : Robust

Expression : Linear prediction, predict()

	Margin	Delta-method Std. Err.	t	P>|t|	[95% Conf.	Interval]
age						
1	56.35919	4.04283	13.94	0.000	48.3663	64.35209
2	58.15064	3.541895	16.42	0.000	51.14813	65.15316
3	65.45899	5.288911	12.38	0.000	55.00252	75.91545
4	73.0763	5.189364	14.08	0.000	62.81665	83.33596
5	71.11323	7.874179	9.03	0.000	55.54555	86.6809

. margin zone

Predictive margins Number of obs = 148
Model VCE : Robust

Expression : Linear prediction, predict()

	Margin	Delta-method Std. Err.	t	P>|t|	[95% Conf.	Interval]
zone						
rural	54.13911	5.359245	10.10	0.000	43.54359	64.73462
periphery	66.06619	3.099216	21.32	0.000	59.93887	72.19351
centro	69.2502	4.402456	15.73	0.000	60.54631	77.95409

Linear regression

Number of obs	=	145
F(10, 134)	=	7.99
Prob > F	=	0.0000
R-squared	=	0.2653
Root MSE	=	983.89

h6cb156	Coef.	Robust Std. Err.	t	P>|t|	[95% Conf.	Interval]
expo	403.6371	188.8072	2.14	0.034	30.20942	777.0648
TRAFFIC						
2	-402.901	218.0084	-1.85	0.067	-834.0836	28.28162
3	-297.5413	296.225	-1.00	0.317	-883.4226	288.3401
4	-421.9678	201.407	-2.10	0.038	-820.3157	-23.61996
age						
2	376.6727	249.0902	1.51	0.133	-115.9842	869.3297
3	593.0005	280.4359	2.11	0.036	38.34713	1147.654
4	1144.328	230.6073	4.96	0.000	688.2274	1600.43
5	1343.359	221.8433	6.06	0.000	904.5918	1782.126
zone						
periphery	511.4882	236.0548	2.17	0.032	44.61298	978.3634
center	511.8462	213.3399	2.40	0.018	89.89711	933.7953
_cons	1113.331	241.9537	4.60	0.000	634.789	1591.873

. margin expo

Predictive margins   Number of obs   =   145

Model VCE   : Robust

Expression   : Linear prediction, predict()

	Margin	Delta-method Std. Err.	t	P>|t|	[95% Conf.	Interval]
expo						
0	1951.926	104.6127	18.66	0.000	1745.02	2158.831
1	2355.563	141.0758	16.70	0.000	2076.539	2634.586

. margin age

Predictive margins   Number of obs   =   145

Model VCE   : Robust

Expression   : Linear prediction, predict()

	Margin	Delta-method Std. Err.	t	P>|t|	[95% Conf.	Interval]
age						
1	1512.815	168.1841	8.99	0.000	1180.176	1845.454
2	1889.488	185.1286	10.21	0.000	1523.336	2255.64
3	2105.816	229.2318	9.19	0.000	1652.435	2559.196
4	2657.144	158.868	16.73	0.000	2342.931	2971.357
5	2856.174	142.5743	20.03	0.000	2574.187	3138.162

. margin zone

Predictive margins Number of obs = 145
Model VCE : Robust

Expression : Linear prediction, predict()

	Margin	Delta-method Std. Err.	t	P>|t|	[95% Conf.	Interval]
zone						
rural	1754.924	153.3237	11.45	0.000	1451.677	2058.172
periphery	2266.413	147.7796	15.34	0.000	1974.13	2558.695
center	2266.771	134.6462	16.84	0.000	2000.464	2533.077

```
Linear regression                        Number of obs     =        145
                                         F(10, 134)        =       6.35
                                         Prob > F          =     0.0000
                                         R-squared         =     0.2311
                                         Root MSE          =     197.22
```

h6cb157	Coef.	Robust Std. Err.	t	P>|t|	[95% Conf.	Interval]
expo	73.62107	37.55034	1.96	0.052	-.6469519	147.8891
TRAFFIC						
2	-71.34957	43.57883	-1.64	0.104	-157.5409	14.84176
3	-47.06082	57.30366	-0.82	0.413	-160.3975	66.27584
4	-80.12059	40.06543	-2.00	0.048	-159.363	-.8781436
age						
2	73.1946	50.60916	1.45	0.150	-26.9015	173.2907
3	86.96761	54.92056	1.58	0.116	-21.65568	195.5909
4	213.977	47.97245	4.46	0.000	119.0959	308.8582
5	232.7514	44.95348	5.18	0.000	143.8413	321.6616
zone						

periphery	94.63448	48.0358	1.97	0.051	-.3719578	189.6409
center	97.55213	43.26824	2.25	0.026	11.9751	183.1292
_cons	241.3807	52.50453	4.60	0.000	137.5359	345.2255

. margin expo

Predictive margins   Number of obs = 145
Model VCE : Robust

Expression : Linear prediction, predict()

	Margin	Delta-method Std. Err.	t	P>|t|	[95% Conf.	Interval]
expo						
0	393.0374	21.62569	18.17	0.000	350.2655	435.8092
1	466.6584	27.61959	16.90	0.000	412.0317	521.2852

. margin age

Predictive margins   Number of obs = 145
Model VCE : Robust

Expression : Linear prediction, predict()

	Margin	Delta-method Std. Err.	t	P>|t|	[95% Conf.	Interval]
age						
1	316.9149	35.37847	8.96	0.000	246.9424	386.8873
2	390.1095	36.1425	10.79	0.000	318.6259	461.5931
3	403.8825	43.0374	9.38	0.000	318.762	489.003
4	530.8919	32.70712	16.23	0.000	466.2029	595.5809
5	549.6663	28.44477	19.32	0.000	493.4075	605.9251

. margin zone

Predictive margins   Number of obs = 145
Model VCE : Robust

Expression : Linear prediction, predict()

	Margin	Delta-method Std. Err.	t	P>|t|	[95% Conf.	Interval]
zone						
rural	355.1166	32.74443	10.85	0.000	290.3538	419.8794

periphery	449.7511	29.24583	15.38	0.000	391.9079	507.5942
center	452.6687	26.57105	17.04	0.000	400.1158	505.2216

Linear regression

Number of obs	=	145
F(10, 134)	=	5.39
Prob > F	=	0.0000
R-squared	=	0.2659
Root MSE	=	293.11

h6cb167	Coef.	Robust Std. Err.	t	P>|t|	[95% Conf.	Interval]
expo	214.1995	52.46807	4.08	0.000	110.4268	317.9722
TRAFFIC						
2	-115.8691	60.98929	-1.90	0.060	-236.4952	4.757142
3	-33.05123	85.81447	-0.39	0.701	-202.7773	136.6748
4	-67.03729	69.15049	-0.97	0.334	-203.8049	69.73033
age						
2	97.44948	72.4669	1.34	0.181	-45.87742	240.7764
3	107.14	74.50419	1.44	0.153	-40.21627	254.4963
4	259.523	69.31847	3.74	0.000	122.4231	396.6228
5	339.0476	86.50269	3.92	0.000	167.9604	510.1349
zone						
periphery	166.2403	69.76337	2.38	0.019	28.26053	304.2201
center	192.9807	67.4581	2.86	0.005	59.56036	326.4011
_cons	350.1465	70.19028	4.99	0.000	211.3223	488.9706

Linear regression

Number of obs	=	148
F(7, 140)	=	6.19
Prob > F	=	0.0000
R-squared	=	0.2254
Root MSE	=	301.2

h6cb167	Coef.	Robust Std. Err.	t	P>|t|	[95% Conf.	Interval]
expo	196.7954	50.70649	3.88	0.000	96.54591	297.0448
age						
2	108.6703	72.7107	1.49	0.137	-35.08269	252.4232
3	137.1516	78.06638	1.76	0.081	-17.18984	291.493
4	252.5148	69.15889	3.65	0.000	115.7839	389.2456

5	302.6809	87.32676	3.47	0.001	130.0312	475.3306
zone						
periphery	150.3902	68.17096	2.21	0.029	15.61255	285.1678
center	163.8336	64.88111	2.53	0.013	35.56012	292.107
_cons	330.2094	71.22858	4.64	0.000	189.3867	471.0321

. margin expo

Predictive margins Number of obs = 148
Model VCE : Robust

Expression : Linear prediction, predict()

	Delta-method					
	Margin	Std. Err.	t	P>|t|	[95% Conf.	Interval]
expo						
0	604.5632	27.7758	21.77	0.000	549.6489	659.4774
1	801.3585	41.55636	19.28	0.000	719.1994	883.5177

. margin age

Predictive margins Number of obs = 148
Model VCE : Robust

Expression : Linear prediction, predict()

	Delta-method					
	Margin	Std. Err.	t	P>|t|	[95% Conf.	Interval]
age						
1	554.2933	49.12022	11.28	0.000	457.18	651.4066
2	662.9636	53.63713	12.36	0.000	556.9201	769.0071
3	691.4449	63.23451	10.93	0.000	566.4269	816.4629
4	806.8081	47.33705	17.04	0.000	713.2202	900.396
5	856.9742	71.13958	12.05	0.000	716.3274	997.621

. margin zone

Predictive margins Number of obs = 148
Model VCE : Robust

Expression : Linear prediction, predict()

	Delta-method				
	Margin	Std. Err.	t	P>|t|	[95% Conf. Interval]

zone						
rural	579.934	51.04378	11.36	0.000	479.0177	680.8503
periphery	730.3242	39.86178	18.32	0.000	651.5153	809.1331
center	743.7676	38.40569	19.37	0.000	667.8374	819.6977

```
Linear regression                               Number of obs     =        145
                                                F(10, 134)        =       8.73
                                                Prob > F          =     0.0000
                                                R-squared         =     0.2287
                                                Root MSE          =     96.705
```

h7cb189	Coef.	Robust Std. Err.	t	P>|t|	[95% Conf.	Interval]
expo	36.55742	17.96467	2.03	0.044	1.026418	72.08841
TRAFFIC						
2	-34.58637	21.60361	-1.60	0.112	-77.31454	8.141808
3	-9.506828	28.16013	-0.34	0.736	-65.20267	46.18901
4	-32.37252	20.00731	-1.62	0.108	-71.94349	7.198443
age						
2	32.18453	20.54643	1.57	0.120	-8.452723	72.82177
3	63.37655	31.7395	2.00	0.048	.6013496	126.1518
4	92.26565	18.77298	4.91	0.000	55.13596	129.3953
5	125.5757	19.41933	6.47	0.000	87.16762	163.9837
zone						
periphery	51.29899	24.55814	2.09	0.039	2.727264	99.87072
center	51.15975	18.81804	2.72	0.007	13.94094	88.37857
_cons	78.902	21.50866	3.67	0.000	36.36163	121.4424

```
Linear regression                               Number of obs     =        148
                                                F(7, 140)         =      11.47
                                                Prob > F          =     0.0000
                                                R-squared         =     0.2163
                                                Root MSE          =       96.9
```

h7cb189	Coef.	Robust Std. Err.	t	P>|t|	[95% Conf.	Interval]
expo	30.29242	16.69332	1.81	0.072	-2.711175	63.29601
age						
2	36.23592	19.8275	1.83	0.070	-2.964104	75.43594

3	66.99371	30.72399	2.18	0.031	6.250734	127.7367
4	90.21277	18.4865	4.88	0.000	53.66397	126.7616
5	128.1575	19.10773	6.71	0.000	90.38049	165.9345
zone						
periphery	42.68891	21.67297	1.97	0.051	-.1597112	85.53754
center	38.79216	16.63459	2.33	0.021	5.90469	71.67964
_cons	74.00781	22.71865	3.26	0.001	29.09182	118.9238

. margin expo

Predictive margins Number of obs = 148
Model VCE : Robust

Expression : Linear prediction, predict()

	Margin	Delta-method Std. Err.	t	P>|t|	[95% Conf.	Interval]
expo						
0	166.3653	8.388882	19.83	0.000	149.7801	182.9506
1	196.6577	13.93369	14.11	0.000	169.1101	224.2054

. margin age

Predictive margins Number of obs = 148
Model VCE : Robust

Expression : Linear prediction, predict()

	Margin	Delta-method Std. Err.	t	P>|t|	[95% Conf.	Interval]
age						
1	122.1043	13.23237	9.23	0.000	95.94321	148.2654
2	158.3402	14.87611	10.64	0.000	128.9294	187.7511
3	189.098	28.54928	6.62	0.000	132.6546	245.5415
4	212.3171	12.95906	16.38	0.000	186.6963	237.9378
5	250.2618	13.352	18.74	0.000	223.8642	276.6594

. margin zone

Predictive margins Number of obs = 148
Model VCE : Robust

Expression : Linear prediction, predict()

	Margin	Delta-method Std. Err.	t	P>|t|	[95% Conf.	Interval]
zone						
rural	148.9706	12.47017	11.95	0.000	124.3164	173.6248
periphery	191.6595	15.37987	12.46	0.000	161.2527	222.0663
center	187.7628	9.980582	18.81	0.000	168.0306	207.4949

Linear regression

Number of obs	=	145
F(10, 134)	=	5.06
Prob > F	=	0.0000
R-squared	=	0.2370
Root MSE	=	1.5406

p5cb99	Coef.	Robust Std. Err.	t	P>|t|	[95% Conf.	Interval]
expo	1.489158	.2726643	5.46	0.000	.9498757	2.028441
TRAFFIC						
2	-.302197	.3588955	-0.84	0.401	-1.01203	.4076357
3	-.4378442	.4348386	-1.01	0.316	-1.297879	.4221908
4	-.8155023	.3603753	-2.26	0.025	-1.528262	-.1027427
age						
2	.1416715	.4012298	0.35	0.725	-.6518912	.9352342
3	-.0417741	.3655788	-0.11	0.909	-.7648254	.6812771
4	.568414	.4146127	1.37	0.173	-.2516178	1.388446
5	.7707242	.5174023	1.49	0.139	-.2526074	1.794056
zone						
periphery	.490574	.3608721	1.36	0.176	-.2231681	1.204316
center	1.317618	.4162427	3.17	0.002	.4943626	2.140874
_cons	2.058756	.3194466	6.44	0.000	1.426946	2.690566

Linear regression

Number of obs	=	148
F(7, 140)	=	5.92
Prob > F	=	0.0000
R-squared	=	0.1956
Root MSE	=	1.5819

p5cb99	Coef.	Robust Std. Err.	t	P>|t|	[95% Conf.	Interval]
expo	1.425361	.266007	5.36	0.000	.8994507	1.951271

age						
2	.2161812	.3952507	0.55	0.585	-.5652506	.997613
3	.0635539	.373057	0.17	0.865	-.6739997	.8011076
4	.5294372	.4203646	1.26	0.210	-.3016461	1.360521
5	.4468662	.5112457	0.87	0.384	-.563894	1.457626
zone						
periphery	.4037902	.3409978	1.18	0.238	-.2703808	1.077961
center	1.045572	.3871338	2.70	0.008	.2801871	1.810956
_cons	1.956186	.3113516	6.28	0.000	1.340627	2.571745

```
Linear regression                                 Number of obs     =        145
                                                  F(6, 138)         =       6.83
                                                  Prob > F          =     0.0000
                                                  R-squared         =     0.2063
                                                  Root MSE          =     1.5484
```

p5cb99	Coef.	Robust Std. Err.	t	P>|t|	[95% Conf.	Interval]
expo	1.496865	.2645067	5.66	0.000	.9738554	2.019875
TRAFFIC						
2	-.2205629	.3631202	-0.61	0.545	-.9385618	.497436
3	-.4499064	.4291295	-1.05	0.296	-1.298426	.3986128
4	-.6682815	.350384	-1.91	0.059	-1.361097	.0245341
zone						
periphery	.6183966	.3494101	1.77	0.079	-.0724933	1.309287
center	1.395406	.3875666	3.60	0.000	.6290687	2.161743
_cons	2.176837	.2974449	7.32	0.000	1.588698	2.764976

```
. margin expo

Predictive margins                                Number of obs     =        145
Model VCE    : Robust

Expression   : Linear prediction, predict()
```

	Margin	Delta-method Std. Err.	t	P>|t|	[95% Conf.	Interval]
expo						
0	2.687277	.157116	17.10	0.000	2.376611	2.997942
1	4.184142	.2066457	20.25	0.000	3.775541	4.592743

. margin zone

Predictive margins   Number of obs = 145
Model VCE : Robust

Expression : Linear prediction, predict()

	Margin	Delta-method Std. Err.	t	P>|t|	[95% Conf.	Interval]
zone						
rural	2.719207	.2816358	9.66	0.000	2.162327	3.276086
periphery	3.337603	.1919295	17.39	0.000	2.9581	3.717106
center	4.114612	.249232	16.51	0.000	3.621805	4.60742

. margin TRAFFIC

Predictive margins   Number of obs = 145
Model VCE : Robust

Expression : Linear prediction, predict()

	Margin	Delta-method Std. Err.	t	P>|t|	[95% Conf.	Interval]
TRAFFIC						
1	3.693388	.1864791	19.81	0.000	3.324663	4.062114
2	3.472825	.3003273	11.56	0.000	2.878987	4.066664
3	3.243482	.3945282	8.22	0.000	2.46338	4.023584
4	3.025107	.2876194	10.52	0.000	2.456396	3.593818

Linear regression   Number of obs = 145
F(10, 134) = 6.47
Prob > F = 0.0000
R-squared = 0.2534
Root MSE = 4.8439

h6cb138	Coef.	Robust Std. Err.	t	P>|t|	[95% Conf.	Interval]
expo	2.38016	.9414715	2.53	0.013	.5180929	4.242226
TRAFFIC						
2	-1.582181	1.075562	-1.47	0.144	-3.709455	.5450924
3	-1.132217	1.225949	-0.92	0.357	-3.55693	1.292496
4	-1.343259	1.196495	-1.12	0.264	-3.709717	1.023199

	Coef.	Std. Err.	t	P>|t|	[95% Conf.	Interval]
age						
2	2.610693	1.230585	2.12	0.036	.1768112	5.044575
3	2.4537	1.063467	2.31	0.023	.3503479	4.557053
4	5.16748	1.182671	4.37	0.000	2.828364	7.506597
5	5.917192	1.618971	3.65	0.000	2.715149	9.119235
zone						
periphery	2.431064	.8968418	2.71	0.008	.6572673	4.204861
center	3.761978	1.028564	3.66	0.000	1.727657	5.796298
_cons	7.384485	1.076582	6.86	0.000	5.255194	9.513777

```
Linear regression                               Number of obs     =        148
                                                F(7, 140)         =       6.98
                                                Prob > F          =     0.0000
                                                R-squared         =     0.2094
                                                Root MSE          =     5.0293
```

h6cb138	Coef.	Robust Std. Err.	t	P>|t|	[95% Conf.	Interval]
expo	2.225479	.8913779	2.50	0.014	.4631767	3.987781
age						
2	2.792033	1.217503	2.29	0.023	.3849631	5.199102
3	2.827037	1.106586	2.55	0.012	.6392569	5.014816
4	5.035581	1.154317	4.36	0.000	2.753433	7.317729
5	4.904025	1.708139	2.87	0.005	1.526941	8.281108
zone						
periphery	2.210956	.9090493	2.43	0.016	.4137169	4.008196
center	3.144945	1.025224	3.07	0.003	1.118022	5.171867
_cons	7.015215	1.070834	6.55	0.000	4.898118	9.132313

```
. margin expo

Predictive margins                              Number of obs     =        148
Model VCE    : Robust

Expression   : Linear prediction, predict()
```

	Margin	Delta-method Std. Err.	t	P>|t|	[95% Conf.	Interval]
expo						
0	12.05857	.6026595	20.01	0.000	10.86708	13.25007
1	14.28405	.6128958	23.31	0.000	13.07233	15.49578

. margin age

Predictive margins Number of obs = 148
Model VCE : Robust

Expression : Linear prediction, predict()

	Margin	Delta-method Std. Err.	t	P>|t|	[95% Conf.	Interval]
age						
1	10.21501	.7841535	13.03	0.000	8.664693	11.76532
2	13.00704	.9433336	13.79	0.000	11.14202	14.87206
3	13.04204	.826083	15.79	0.000	11.40883	14.67525
4	15.25059	.8537561	17.86	0.000	13.56267	16.93851
5	15.11903	1.510111	10.01	0.000	12.13346	18.1046

. margin zone

Predictive margins Number of obs = 148
Model VCE : Robust

Expression : Linear prediction, predict()

	Margin	Delta-method Std. Err.	t	P>|t|	[95% Conf.	Interval]
zone						
rural	11.11434	.666221	16.68	0.000	9.797181	12.43149
periphery	13.32529	.6125269	21.75	0.000	12.11429	14.53629
center	14.25928	.8096306	17.61	0.000	12.6586	15.85996

```
Linear regression                               Number of obs     =        145
                                                F(10, 134)        =       7.63
                                                Prob > F          =     0.0000
                                                R-squared         =     0.2776
                                                Root MSE          =     9.8951
```

h6cb153	Coef.	Robust Std. Err.	t	P>|t|	[95% Conf.	Interval]
expo	5.361128	1.866835	2.87	0.005	1.668854	9.053402
TRAFFIC						
2	-3.332623	2.210062	-1.51	0.134	-7.70374	1.038495

	Coef.	Std. Err.	t	P>|t|	[95% Conf.	Interval]
3	-2.329193	2.749499	-0.85	0.398	-7.767223	3.108837
4	-3.990321	2.183447	-1.83	0.070	-8.308799	.3281574
age						
2	4.827615	2.630033	1.84	0.069	-.3741322	10.02936
3	4.661801	2.454807	1.90	0.060	-.1933803	9.516983
4	10.49192	2.463995	4.26	0.000	5.618571	15.36528
5	13.37695	2.857199	4.68	0.000	7.725907	19.02799
zone						
periphery	6.063817	2.120311	2.86	0.005	1.870212	10.25742
center	7.934118	2.26047	3.51	0.001	3.463303	12.40493
_cons	13.28284	2.258967	5.88	0.000	8.814995	17.75068

Linear regression

Number of obs	=	148
F(7, 140)	=	7.96
Prob > F	=	0.0000
R-squared	=	0.2281
Root MSE	=	10.314

h6cb153	Coef.	Robust Std. Err.	t	P>|t|	[95% Conf.	Interval]
expo	4.985152	1.813895	2.75	0.007	1.398985	8.571319
age						
2	5.066365	2.641773	1.92	0.057	-.1565627	10.28929
3	5.539205	2.532187	2.19	0.030	.5329354	10.54548
4	10.20272	2.421161	4.21	0.000	5.415956	14.98949
5	11.22776	3.044645	3.69	0.000	5.208337	17.24719
zone						
periphery	5.442766	2.064541	2.64	0.009	1.361058	9.524474
center	6.372207	2.207007	2.89	0.005	2.008835	10.73558
_cons	12.46988	2.257677	5.52	0.000	8.006336	16.93343

Linear regression

Number of obs	=	148
F(7, 140)	=	10.01
Prob > F	=	0.0000
R-squared	=	0.2898
Root MSE	=	.20943

t4cdd2378	Coef.	Robust Std. Err.	t	P>|t|	[95% Conf.	Interval]

	Coef.	Std. Err.	t	P>|t|	[95% Conf.	Interval]
expo	.2222409	.0374569	5.93	0.000	.1481866	.2962951
age						
2	.053259	.0548866	0.97	0.334	-.0552547	.1617727
3	.001222	.057958	0.02	0.983	-.1133642	.1158081
4	.1091835	.046896	2.33	0.021	.0164676	.2018993
5	.0815258	.0707301	1.15	0.251	-.0583114	.221363
zone						
periphery	.0894393	.0470333	1.90	0.059	-.0035481	.1824267
center	.0313295	.0476966	0.66	0.512	-.0629692	.1256282
_cons	.3264603	.0477733	6.83	0.000	.2320099	.4209107

. margins expo

Predictive margins Number of obs = 148
Model VCE : Robust

Expression : Linear prediction, predict()

	Margin	Delta-method Std. Err.	t	P>|t|	[95% Conf.	Interval]
expo						
0	.4255536	.0233683	18.21	0.000	.3793532	.471754
1	.6477945	.0273007	23.73	0.000	.5938195	.7017695

Table S3. Regression final models: main predictors for the dependent variables (chemicals)

```
Linear regression                               Number of obs     =        148
                                                F(3, 144)         =      22.18
                                                Prob > F          =     0.0000
                                                R-squared         =     0.2801
                                                Root MSE          =     3.3955
```

Σ10(PCDFs)	Coef.	Robust Std. Err.	t	P>|t|	[95% Conf.	Interval]
expo	4.240739	.5460099	7.77	0.000	3.161509	5.319969
zone						
periphery	1.964621	.6605111	2.97	0.003	.6590716	3.270171
center	2.610876	.728328	3.58	0.000	1.171281	4.050471
_cons	6.584816	.6193642	10.63	0.000	5.360596	7.809036

```
. margins expo

Predictive margins                              Number of obs     =        148
Model VCE    : Robust

Expression   : Linear prediction, predict()
```

---------------------- --

. margins zone

Predictive margins Number of obs = 148
Model VCE : Robust

Expression : Linear prediction, predict()

	Margin	Delta-method Std. Err.	t	P>|t|	[95% Conf.	Interval]
zone						
rural	8.733839	.5082375	17.18	0.000	7.72927	9.738409
periphery	10.69846	.4059096	26.36	0.000	9.89615	11.50077
center	11.34472	.5219004	21.74	0.000	10.31314	12.37629

Linear regression

Number of obs	=	148
F(7, 140)	=	5.98
Prob > F	=	0.0000
R-squared	=	0.2434
Root MSE	=	2.0183

TEQTOT	Coef.	Robust Std. Err.	t	P>|t|	[95% Conf.	Interval]
expo	1.650887	.3421204	4.83	0.000	.9744962	2.327277
age						
2	.4963062	.5114359	0.97	0.334	-.5148302	1.507442
3	.7085131	.5099556	1.39	0.167	-.2996964	1.716723
4	1.466471	.4384928	3.34	0.001	.5995469	2.333394
5	1.61097	.5998703	2.69	0.008	.4249943	2.796946
zone						
periphery	1.161629	.4489941	2.59	0.011	.2739433	2.049314
center	1.021906	.4513518	2.26	0.025	.1295587	1.914252
_cons	4.007499	.4901674	8.18	0.000	3.038412	4.976586

. margins expo

Predictive margins   Number of obs = 148
Model VCE : Robust

Expression : Linear prediction, predict()

	Margin	Delta-method Std. Err.	t	P>|t|	[95% Conf.	Interval]
expo						
0	5.697862	.2326488	24.49	0.000	5.237902	6.157821
1	7.348748	.2436488	30.16	0.000	6.867041	7.830455

. margins age

Predictive margins   Number of obs = 148
Model VCE : Robust

Expression : Linear prediction, predict()

	Margin	Delta-method Std. Err.	t	P>|t|	[95% Conf.	Interval]

age						
1	5.724001	.3162098	18.10	0.000	5.098837	6.349164
2	6.220307	.3986645	15.60	0.000	5.432126	7.008488
3	6.432514	.4088179	15.73	0.000	5.624259	7.240769
4	7.190471	.3102942	23.17	0.000	6.577003	7.803939
5	7.194971	.4902271	14.96	0.000	6.365765	8.304176

. margins zone

Predictive margins Number of obs = 148
Model VCE : Robust

Expression : Linear prediction, predict()

	Margin	Delta-method Std. Err.	t	P>|t|	[95% Conf.	Interval]
zone						
rural	5.654556	.3573007	15.83	0.000	4.948153	6.360958
periphery	6.816185	.264024	25.82	0.000	6.294195	7.338174
center	6.676461	.2598597	25.69	0.000	6.162705	7.190218

Linear regression

Number of obs = 148
F(7, 140) = 7.80
Prob > F = 0.0000
R-squared = 0.2828
Root MSE = 1.1637

PCDDs+PCDFs	Coef.	Robust Std. Err.	t	P>|t|	[95% Conf. Interval]	
expo	1.196416	.1996235	5.99	0.000	.8017501	1.591083
age						
2	.2496621	.3221671	0.77	0.440	-.3872795	.8866038
3	.2554073	.2854605	0.89	0.372	-.3089634	.819778
4	.7430466	.2495666	2.98	0.003	.2496399	1.236453
5	.8181089	.3498041	2.34	0.021	.1265274	1.50969
zone						
periphery	.6170131	.2466734	2.50	0.014	.1293265	1.1047
center	.501411	.2460473	2.04	0.043	.0149623	.9878597
_cons	2.500125	.28231	8.86	0.000	1.941983	3.058267

. margins expo

Predictive margins   Number of obs = 148
Model VCE : Robust

Expression : Linear prediction, predict()

	Margin	Delta-method Std. Err.	t	P>|t|	[95% Conf.	Interval]
expo						
0	3.34198	.1358736	24.60	0.000	3.07335	3.610609
1	4.538396	.140512	32.30	0.000	4.260596	4.816196

. margins age

Predictive margins   Number of obs = 148
Model VCE : Robust

Expression : Linear prediction, predict()

	Margin	Delta-method Std. Err.	t	P>|t|	[95% Conf.	Interval]
age						
1	3.560087	.1880925	18.93	0.000	3.188218	3.931956
2	3.809749	.2622722	14.53	0.000	3.291223	4.328275
3	3.815494	.2180037	17.50	0.000	3.384489	4.246499
4	4.303133	.171972	25.02	0.000	3.963135	4.643131
5	4.378196	.2835157	15.44	0.000	3.81767	4.938721

. margins zone

Predictive margins   Number of obs = 148
Model VCE : Robust

Expression : Linear prediction, predict()

	Margin	Delta-method Std. Err.	t	P>|t|	[95% Conf.	Interval]
zone						
rural	3.494602	.1896643	18.43	0.000	3.119626	3.869579
periphery	4.111616	.1543808	26.63	0.000	3.806397	4.416835
center	3.996013	.1494556	26.74	0.000	3.700532	4.291495

Linear regression   Number of obs = 148
                                                         F(3, 144) = 4.12

Prob > F	= 0.0078
R-squared	= 0.0427
Root MSE	= 13.411

Σ7(PCDDs)	Coef.	Robust Std. Err.	t	P>|t|	[95% Conf.	Interval]
expo	4.199966	2.008816	2.09	0.038	.22939	8.170542
zone						
periphery	4.143039	2.088743	1.98	0.049	.0144824	8.271596
center	7.13815	2.733741	2.61	0.010	1.734705	12.5416
_cons	20.07002	1.975219	10.16	0.000	16.16585	23.97419

. margins expo

Predictive margins Number of obs = 148

Model VCE : Robust

Expression : Linear prediction, predict()

	Margin	Delta-method Std. Err.	t	P>|t|	[95% Conf. Interval]	
expo						
0	24.36488	1.612333	15.11	0.000	21.17799	27.55178
1	28.56485	1.363092	20.96	0.000	25.87059	31.2591

. margins

Predictive margins Number of obs = 148

Model VCE : Robust

Expression : Linear prediction, predict()

	Margin	Delta-method Std. Err.	t	P>|t|	[95% Conf.	Interval]
zone						
rural	22.19838	1.5048	14.75	0.000	19.22403	25.17274
periphery	26.34142	1.407171	18.72	0.000	23.56004	29.1228
center	29.33654	2.383381	12.31	0.000	24.6256	34.04747

Linear regression Number of obs = 148

F(7, 140) = 9.64

Prob > F	=	0.0000
R-squared	=	0.2550
Root MSE	=	20.769

Σ6(NDL-PCBs)	Coef.	Robust Std. Err.	t	P>|t|	[95% Conf.	Interval]
expo	10.13683	3.66393	2.77	0.006	2.893038	17.38061
age						
2	10.94224	5.19057	2.11	0.037	.6802007	21.20427
3	13.61166	5.353894	2.54	0.012	3.026729	24.1966
4	22.19227	4.715395	4.71	0.000	12.86969	31.51486
5	25.95912	5.819012	4.46	0.000	14.45462	37.46362
zone						
periphery	11.37369	4.215146	2.70	0.008	3.040118	19.70726
center	13.22177	4.292901	3.08	0.002	4.734472	21.70906
_cons	25.2096	4.666081	5.40	0.000	15.9845	34.43469

. margins expo

Predictive margins — Number of obs = 148

Model VCE : Robust

Expression : Linear prediction, predict()

	Margin	Delta-method Std. Err.	t	P>|t|	[95% Conf.	Interval]
expo						
0	48.56174	2.307624	21.04	0.000	43.99945	53.12404
1	58.69857	2.681325	21.89	0.000	53.39744	63.99969

. margins zone

Predictive margins — Number of obs = 148

Model VCE : Robust

Expression : Linear prediction, predict()

	Margin	Delta-method Std. Err.	t	P>|t|	[95% Conf.	Interval]
zone						
rural	44.01857	2.970053	14.82	0.000	38.14662	49.89053
periphery	55.39226	2.777569	19.94	0.000	49.90086	60.88366
center	57.24034	2.993552	19.12	0.000	51.32193	63.15875

. margins age

Predictive margins Number of obs = 148
Model VCE : Robust

Expression : Linear prediction, predict()

	Delta-method					
	Margin	Std. Err.	t	P>|t|	[95% Conf.	Interval]
age						
1	40.02658	3.364355	11.90	0.000	33.37507	46.67809
2	50.96881	4.006257	12.72	0.000	43.04823	58.8894
3	53.63824	4.343992	12.35	0.000	45.04994	62.22655
4	62.21885	3.26882	19.03	0.000	55.75622	68.68148
5	65.98569	4.676881	14.11	0.000	56.73925	75.23214

Linear regression

Number of obs = 145
F(10, 134) = 5.56
Prob > F = 0.0000
R-squared = 0.2915
Root MSE = 3583.9

		Robust				
Σ12(DL-PCBs)	Coef.	Std. Err.	t	P>|t|	[95% Conf.	Interval]
expo	2715.001	634.8811	4.28	0.000	1459.317	3970.685
TRAFFIC						
2	-1465.834	757.4242	-1.94	0.055	-2963.887	32.21887
3	-81.21549	1127.125	-0.07	0.943	-2310.472	2148.041
4	-1132.4	859.0274	-1.32	0.190	-2831.406	566.6067
age						
2	1054.206	856.7375	1.23	0.221	-640.2712	2748.684
3	1407.978	818.9842	1.72	0.088	-211.8303	3027.786
4	3647.689	843.3186	4.33	0.000	1979.751	5315.626
5	4366.697	1143.291	3.82	0.000	2105.467	6627.927
zone						
periphery	1986.73	807.5895	2.46	0.015	389.4582	3584.001
center	2559.753	886.5435	2.89	0.005	806.3249	4313.182
_cons	5261.326	845.6511	6.22	0.000	3588.775	6933.876

. margins expo, atmeans

	Margin	Delta-method Std. Err.	t	P>|t|	[95% Conf.	Interval]
expo						
0	8370.001	357.1656	23.43	0.000	7663.59	9076.413
1	11085	496.7692	22.31	0.000	10102.48	12067.53

.

Adjusted predictions Number of obs = 145

Model VCE : Robust

	Margin	Delta-method Std. Err.	t	P>|t|	[95% Conf.	Interval]
TRAFFIC_new						
1	10375.42	418.217	24.81	0.000	9548.264	11202.59
2	8909.59	616.7296	14.45	0.000	7689.807	10129.37
3	10294.21	1088.43	9.46	0.000	8141.484	12446.93
4	9243.025	730.887	12.65	0.000	7797.458	10688.59

. margins age, atmeans

	Margin	Delta-method Std. Err.	t	P>|t|	[95% Conf.	Interval]
age_new						
1	7825.945	579.9719	13.49	0.000	6678.861	8973.028
2	8880.151	631.8396	14.05	0.000	7630.483	10129.82
3	9233.923	614.0647	15.04	0.000	8019.41	10448.44
4	11473.63	611.4228	18.77	0.000	10264.35	12682.92
5	12192.64	964.0605	12.65	0.000	10285.9	14099.39

. margins zone, atmeans

	Margin	Delta-method Std. Err.	t	P>|t|	[95% Conf.	Interval]
zone						
rural	8012.891	652.5565	12.28	0.000	6722.249	9303.534
periphery	9999.621	467.8407	21.37	0.000	9074.314	10924.93

center	10572.64	563.5925	18.76	0.000	9457.957	11687.33

```
Linear regression                               Number of obs     =        148
                                                F(7, 140)         =       3.56
                                                Prob > F          =     0.0015
                                                R-squared         =     0.1567
                                                Root MSE          =     1.0549
```

DLPCB	Coef.	Robust Std. Err.	t	P>|t|	[95% Conf.	Interval]
expo	.4554145	.1778316	2.56	0.011	.1038319	.8069971
age						
2	.2728644	.2374752	1.15	0.253	-.1966367	.7423656
3	.4683269	.2663511	1.76	0.081	-.0582636	.9949174
4	.7401362	.2425237	3.05	0.003	.2606537	1.219619
5	.7999177	.325927	2.45	0.015	.1555425	1.444293
zone						
periphery	.5499758	.2388287	2.30	0.023	.0777986	1.022153
center	.522746	.2505983	2.09	0.039	.0272996	1.018192
_cons	1.487091	.2602625	5.71	0.000	.9725381	2.001644

```
Linear regression                               Number of obs     =        145
                                                F(10, 134)        =       4.80
                                                Prob > F          =     0.0000
                                                R-squared         =     0.2835
                                                Root MSE          =       1.99
```

TEQTOT	Coef.	Robust Std. Err.	t	P>|t|	[95% Conf.	Interval]
expo	1.884125	.3505773	5.37	0.000	1.190744	2.577506
TRAFFIC						
2	-.3584627	.4331719	-0.83	0.409	-1.215201	.4982759
3	.8688351	.7948675	1.09	0.276	-.7032742	2.440944
4	-.6009341	.4432371	-1.36	0.177	-1.47758	.2757116
age						
2	.3513077	.5317848	0.66	0.510	-.7004699	1.403085
3	.4550213	.4765312	0.95	0.341	-.4874745	1.397517
4	1.421997	.4305997	3.30	0.001	.5703457	2.273648
5	1.899608	.6289895	3.02	0.003	.6555768	3.14364

	Coef.	Robust Std. Err.	t	P>|t|	[95% Conf.	Interval]
zone						
periphery	1.087528	.4500045	2.42	0.017	.1974975	1.977559
center	1.240003	.4827775	2.57	0.011	.2851533	2.194853
_cons	4.008769	.5023664	7.98	0.000	3.015175	5.002362

Linear regression

Number of obs	=	148
F(7, 140)	=	5.98
Prob > F	=	0.0000
R-squared	=	0.2434
Root MSE	=	2.0183

TEQTOT	Coef.	Robust Std. Err.	t	P>|t|	[95% Conf.	Interval]
expo	1.650887	.3421204	4.83	0.000	.9744962	2.327277
age						
2	.4963062	.5114359	0.97	0.334	-.5148302	1.507442
3	.7085131	.5099556	1.39	0.167	-.2996964	1.716723
4	1.466471	.4384928	3.34	0.001	.5995469	2.333394
5	1.61097	.5998703	2.69	0.008	.4249943	2.796946
zone						
periphery	1.161629	.4489941	2.59	0.011	.2739433	2.049314
centro	1.021906	.4513518	2.26	0.025	.1295587	1.914252
_cons	4.007499	.4901674	8.18	0.000	3.038412	4.976586

Linear regression

Number of obs	=	145
F(10, 134)	=	6.22
Prob > F	=	0.0000
R-squared	=	0.3012
Root MSE	=	1.1729

PCDDs+PCDFs	Coef.	Robust Std. Err.	t	P>|t|	[95% Conf.	Interval]
expo	1.285102	.2050734	6.27	0.000	.8795024	1.690701
TRAFFIC						
2	-.021201	.2697194	-0.08	0.937	-.554659	.512257
3	.3207061	.4489922	0.71	0.476	-.5673223	1.208735
4	-.3400559	.2596418	-1.31	0.193	-.8535822	.1734704
age						
2	.1858149	.338367	0.55	0.584	-.483416	.8550458
3	.1742773	.2760581	0.63	0.529	-.3717175	.7202721

	Coef.	Std. Err.	t	P>|t|	[95% Conf.	Interval]
4	.7214264	.2474192	2.92	0.004	.2320743	1.210778
5	.9336167	.3751455	2.49	0.014	.1916443	1.675589
zone						
periphery	.5823722	.2477038	2.35	0.020	.0924572	1.072287
center	.6048836	.2650166	2.28	0.024	.0807269	1.12904
_cons	2.488176	.2875577	8.65	0.000	1.919436	3.056915

Linear regression

Number of obs	=	148
F(7, 140)	=	7.80
Prob > F	=	0.0000
R-squared	=	0.2828
Root MSE	=	1.1637

PCDDs+PCDFs	Coef.	Robust Std. Err.	t	P>|t|	[95% Conf.	Interval]
expo	1.196416	.1996235	5.99	0.000	.8017501	1.591083
age						
2	.2496621	.3221671	0.77	0.440	-.3872795	.8866038
3	.2554073	.2854605	0.89	0.372	-.3089634	.819778
4	.7430466	.2495666	2.98	0.003	.2496399	1.236453
5	.8181089	.3498041	2.34	0.021	.1265274	1.50969
zone						
periphery	.6170131	.2466734	2.50	0.014	.1293265	1.1047
center	.501411	.2460473	2.04	0.043	.0149623	.9878597
_cons	2.500125	.28231	8.86	0.000	1.941983	3.058267

Linear regression

Number of obs	=	145
F(10, 134)	=	2.90
Prob > F	=	0.0026
R-squared	=	0.2213
Root MSE	=	1.0095

DLPCB	Coef.	Robust Std. Err.	t	P>|t|	[95% Conf.	Interval]
expo	.6006834	.1814074	3.31	0.001	.2418912	.9594756
TRAFFIC						
2	-.3358215	.2039674	-1.65	0.102	-.7392335	.0675906
3	.5454471	.3741204	1.46	0.147	-.1944979	1.285392
4	-.2622868	.2392523	-1.10	0.275	-.7354863	.2109126

age						
2	.1901598	.2398843	0.79	0.429	-.2842895	.664609
3	.2952296	.2399748	1.23	0.221	-.1793988	.769858
4	.7173511	.2376832	3.02	0.003	.2472551	1.187447
5	.9731476	.3215777	3.03	0.003	.337123	1.609172
zone						
periphery	.5102529	.2419539	2.11	0.037	.0317103	.9887956
center	.6370299	.2634636	2.42	0.017	.1159448	1.158115
_cons	1.500167	.2673036	5.61	0.000	.9714875	2.028847

Linear regression

Number of obs	=	148
F(7, 140)	=	3.56
Prob > F	=	0.0015
R-squared	=	0.1567
Root MSE	=	1.0549

DLPCB	Coef.	Robust Std. Err.	t	P>|t|	[95% Conf. Interval]	
expo	.4554145	.1778316	2.56	0.011	.1038319	.8069971
age						
2	.2728644	.2374752	1.15	0.253	-.1966367	.7423656
3	.4683269	.2663511	1.76	0.081	-.0582636	.9949174
4	.7401362	.2425237	3.05	0.003	.2606537	1.219619
5	.7999177	.325927	2.45	0.015	.1555425	1.444293
zone						
periphery	.5499758	.2388287	2.30	0.023	.0777986	1.022153
center	.522746	.2505983	2.09	0.039	.0272996	1.018192
_cons	1.487091	.2602625	5.71	0.000	.9725381	2.001644

. margins expo

Predictive margins — Number of obs = 148

Model VCE : Robust

Expression : Linear prediction, predict()

	Margin	Delta-method Std. Err.	t	P>|t|	[95% Conf.	Interval]
expo						
0	2.351648	.1183069	19.88	0.000	2.117749	2.585547
1	2.807063	.1296664	21.65	0.000	2.550705	3.06342

. margins age

Predictive margins Number of obs = 148
Model VCE : Robust

Expression : Linear prediction, predict()

	Margin (Delta-method)	Std. Err.	t	P>|t|	[95% Conf.	Interval]
age						
1	2.147354	.1679985	12.78	0.000	1.815212	2.479496
2	2.420219	.1652541	14.65	0.000	2.093503	2.746935
3	2.615681	.2119069	12.34	0.000	2.19673	3.034633
4	2.887491	.1739	16.60	0.000	2.543681	3.2313
5	2.947272	.2727693	10.80	0.000	2.407993	3.486552

. margins zone

Predictive margins Number of obs = 148
Model VCE : Robust

Expression : Linear prediction, predict()

	Margin (Delta-method)	Std. Err.	t	P>|t|	[95% Conf.	Interval]
zone						
rural	2.152953	.1968606	10.94	0.000	1.76375	2.542157
periphery	2.702929	.1308215	20.66	0.000	2.444288	2.96157
center	2.675699	.1450668	18.44	0.000	2.388895	2.962504
